# Intensive care unit depth of sleep: proof of concept of a simple electroencephalography index in the non-sedated

**DOI:** 10.1186/cc13823

**Published:** 2014-04-09

**Authors:** Laurens Reinke, Johannes H van der Hoeven, Michel JAM van Putten, Willem Dieperink, Jaap E Tulleken

**Affiliations:** 1 Department of Critical Care, University Medical Center Groningen, University of Groningen, Hanzeplein 1, Groningen 9700RB, The Netherlands; 2 University of Twente, MIRA Institute for Biomedical Technology and Technical Medicine, NL-7500 AE Enschede, the Netherlands; 3 Department of Neurology, University Medical Center Groningen, University of Groningen, Hanzeplein 1, Groningen 9700RB, the Netherlands

## Abstract

**Introduction:**

Intensive care unit (ICU) patients are known to experience severely disturbed sleep, with possible detrimental effects on short- and long- term outcomes. Investigation into the exact causes and effects of disturbed sleep has been hampered by cumbersome and time consuming methods of measuring and staging sleep. We introduce a novel method for ICU depth of sleep analysis, the ICU depth of sleep index (IDOS index), using single channel electroencephalography (EEG) and apply it to outpatient recordings. A proof of concept is shown in non-sedated ICU patients.

**Methods:**

Polysomnographic (PSG) recordings of five ICU patients and 15 healthy outpatients were analyzed using the IDOS index, based on the ratio between gamma and delta band power. Manual selection of thresholds was used to classify data as either wake, sleep or slow wave sleep (SWS). This classification was compared to visual sleep scoring by Rechtschaffen & Kales criteria in normal outpatient recordings and ICU recordings to illustrate face validity of the IDOS index.

**Results:**

When reduced to two or three classes, the scoring of sleep by IDOS index and manual scoring show high agreement for normal sleep recordings. The obtained overall agreements, as quantified by the kappa coefficient, were 0.84 for sleep/wake classification and 0.82 for classification into three classes (wake, non-SWS and SWS). Sensitivity and specificity were highest for the wake state (93% and 93%, respectively) and lowest for SWS (82% and 76%, respectively). For ICU recordings, agreement was similar to agreement between visual scorers previously reported in literature.

**Conclusions:**

Besides the most satisfying visual resemblance with manually scored normal PSG recordings, the established face-validity of the IDOS index as an estimator of depth of sleep was excellent. This technique enables real-time, automated, single channel visualization of depth of sleep, facilitating the monitoring of sleep in the ICU.

## Introduction

Sleep is a dynamic, complex and vital state of human physiology [1]. Sleep is essential to life, and is thought to be restorative, conservative, adaptive, thermoregulatory and have memory consolidative functions [2]. Unfortunately, sleep deprivation is placed among the most common stressors experienced during critical illness [3]. In intensive care unit (ICU) clinical practice it is assumed that sleep is important in the recovery process of the critically ill ICU patients and there are strong indications that ICU delirium and sleep deprivation are closely intertwined [4,5].

In critically ill patients disturbance of sleep is very common but poorly understood. Polysomnographic (PSG) studies in both mechanically ventilated and non-ventilated critical care patients demonstrate that these sleep disturbances are characterized by severe fragmentation by frequent arousals and awakenings [6,7]. Sleep architecture is disrupted with a dominance of stage-1 and stage-2 non rapid eye movement (NREM) sleep with reduced deeper phases of sleep (slow wave sleep (SWS) and rapid eye movement (REM) sleep). For patients in the ICU, sleep traverses the day-night interface, with approximately half of the total sleep time occurring during the day. Total sleep time averages between 2.1 and 8.8 hours of fragmented sleep [8-11].

Spectral composition of the electroencephalogram (EEG) varies as the brain transitions from one sleep stage to the next and each sleep stage has its unique spectral composition. Originally, these sleep stages were defined by their unique spectral composition and physiological relevance. With the aberrant EEG states observed in ICU patients, classification is often hampered by the rules of visual analysis, which have to our knowledge never been validated in the critically ill. Drouot *et al*. recently reported that certain brain states could not be classified according to Rechtschaffen and Kales (R&K) criteria [12], advocating inclusion of two new alternative states for ICU sleep research. The finding of contradictory EEG and electromyography (EMG) activity has been reported previously in other ICU sleep research, often finding sleep-like delta activity in otherwise alert and communicative patients [8,12,13]. Very recently Watson *et al*. reported that 85% of all sleep observed in 37 mechanically ventilated ICU patients was of an atypical nature [14].

The increasing interest in the beneficial effects of sleep and the detrimental effects of disturbed sleep has led to a call for automated sleep analysis since manually scoring sleep is both expensive and time-consuming and requires trained personnel [15,16]. Fortunately, sleep can be analyzed, as is increasingly the case, by studying objective properties of the EEG avoiding subjective interpretation [17,18].

We introduce a novel method to determine depth of sleep from a single EEG channel. It estimates the most relevant aspect of sleep, that is, depth of sleep over time, and can be used to determine quality and quantity of sleep, specifically in ICU sleep research. We call this method ‘ICU Depth Of Sleep’, or IDOS. The face-validity and physiological basis of the new IDOS index is illustrated by comparing its application in healthy individuals with R&K visual analysis. This required manually classifying the IDOS index into discrete sleep stages. The method was also compared to R&K classification in a small sample of ICU patients as a proof of concept.

## Materials and methods

### ICU depth of sleep index

**Figure 1:**
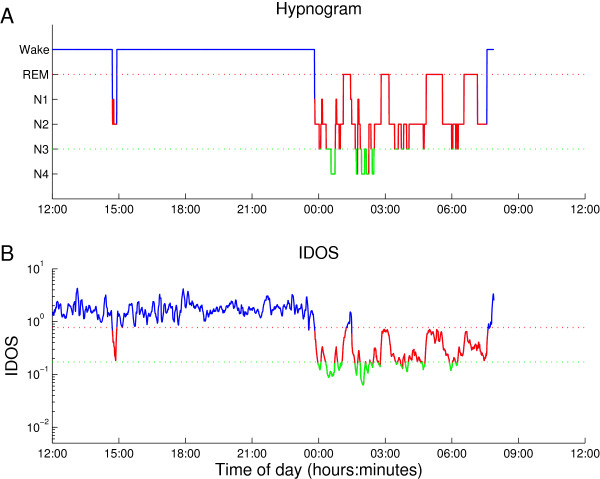
**Hypnogram and IDOS index of the same recording.** To illustrate the resemblance of the visually scored R&K **(1A)** hypnogram and IDOS index **(1B)**, an example is shown. This recording shows normal sleep and is one of the 15 datasets used for further comparison. The hypnogram resulting from R&K analysis **(1A)** shows a long period of wake EEG with occasional transitional sleep. Towards midnight the EEG transitions to increasingly deep stages of sleep, before gradually resurfacing to shallow stages of sleep. This process is repeated roughly four times, known as ultradian rhythm. The IDOS index **(1B)** of the same recording shows similar transitions of depth of sleep. Simple linear thresholds are manually selected to define the transition from wake (blue) to sleep (red) and the transition to SWS (green). The same method of classification has been applied to all fifteen non-ICU recordings and all five ICU recordings. EEG, electroencephalogram; IDOS, ICU Depth of Sleep; R&K, Rechtschaffen and Kales; SWS, slow wave sleep.

**Figure 2:**
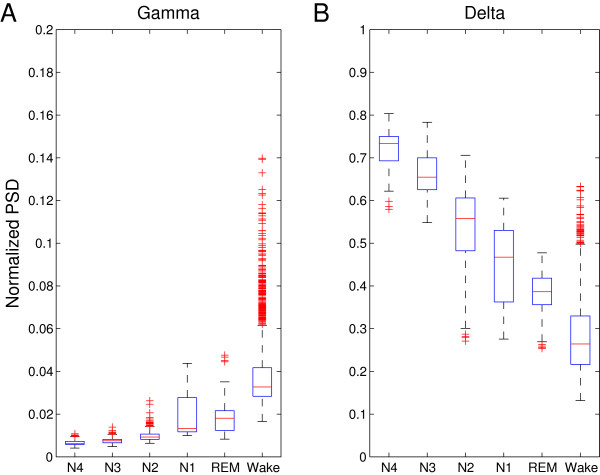
**Boxplots of the gamma (2A) and delta (2B) PSD.** The power spectral densities for the R&K stages from the same recording used for Figure 1 (one of the 15 recordings of normal sleep) show the unique spectral composition of different sleep stages and the wake EEG. The central mark of the boxplots represents the median value; the boxes extend to the 25th and 75th percentile. The confidence intervals extend to a maximum of +/- 2.7 SD. All points outside this range are displayed as outliers, as red plusses. As sleep deepens (towards N4) delta PSD increases at the cost of gamma PSD, with the exception of REM sleep. EEG, electroencephalogram; R&K, Rechtschaffen and Kales; PSD, power spectral density; REM, rapid eye movement; SD, standard deviation.

Delta power activity is known to decline during the night and to increase as sleep deepens during individual sleep cycles. Conversely, high frequency activity increases as the brain shifts towards wakefulness, particularly in the gamma frequency band [19]. This global property of the EEG during sleep has led to the use of power ratios in sleep analysis, mainly between low frequency and high frequency bands [15,20,21]. The gamma and delta band powers for the individual stages of sleep as defined by R&K for a representative recording of normal sleep are given in Figure 1. Dividing gamma power by delta power for individual epochs results in a temporal estimate of depth of sleep, the IDOS index, visually similar to the hypnogram gained by R&K analysis. This visual resemblance is illustrated in Figure 2, where the index is calculated for the same recording as the one used in Figure 1.

A bandpass-filter was applied to remove high-frequency noise and low-frequency drifts and artefacts (caused by breathing, sweating) using a 16th order Butterworth filter between 0.5 Hz and 48 Hz on the single channel EEG data. To stay true to the simplicity and real-time performance of the method, no manual techniques of artefact removal were applied. Discrete short-time Fourier transformation was applied using a two second Hamming window with 50% overlap, resulting in a frequency resolution of 0.5 Hz. Spectral densities were then smoothed using a 240 second moving average square window. The delta (0.5 to 4 Hz), theta (4 to 7 Hz), alpha (7 to 12 Hz), beta (12 to 30 Hz) and gamma (30 to 48 Hz) powers were obtained by combination of the power spectral densities (PSDs) of the corresponding 0.5 Hz bins. The power in each frequency band was normalized by calculating the power in each frequency band relative to total power in the range 0.5 to 48 Hz.

### Patients and controls

Five patients admitted to the ICU of the department of Critical Care of the University Medical Center Groningen, Groningen, The Netherlands were enrolled in our study. Informed consent was obtained from each patient. The local medical ethics committee (Medical Ethical Committee of the University Medical Center Groningen (METc UMCG), research project number 2012.185) reviewed and approved the study protocols. Patients received all aspects of normal care during ICU stay according to standardized protocols. PSG recordings of the controls were obtained from our outpatient clinical database. Fifteen recordings were evaluated as exhibiting sleep without relevant abnormalities and were selected for further use. These patients were referred to the sleep laboratory for suspected sleep apnea (12, 80%), restless legs syndrome (1, 6.66%), insomnia (1, 6.66%) or chronic fatigue syndrome (1, 6.66%).

### Polysomnography

Polysomnography (PSG) sleep recording included a six channel EEG, two channel electro-oculogram (EOG) of ocular movements and an EMG of the left and right masseter muscle or the submental muscles. Furthermore, pulse oxymetry and a 12-lead electrocardiography (ECG) were performed. EEG-electrodes were placed according to the international 10–20 system with Ag/AgCl electrodes, sharing the same reference. EEG, EMG, EOG and ECG were sampled at 256 Hz using either an Embla® A10 (Medcare, Reykjavik, Iceland) or Morpheus® (Micromed, Mogliano Veneto, Italy) digital recorder. The patients' skin was prepared according to standard techniques. In controls, additional polygraphic sensors were placed depending on the clinical question, for example, tibial muscle EMG electrodes to detect restless legs. These additional sensors were not used in the ICU recordings. All recordings in the control group were done in an ambulatory setting starting and ending between 10 am and 5 pm. All recordings were visually scored by the same clinical neurophysiologist using standard R&K criteria, in 30-second epochs [22]. The temporal classification of sleep and wake stages was done by visual interpretation of individual epochs in the software environment Brain RT (OSG, Rumst, Belgium). Among other PSG-derived data, total sleep time (TST), sleep efficiency and percentage spent in each sleep stage were determined to describe quality and quantity of sleep. PSG was performed for a minimum of 24 hours and up to 72 hours depending on the patient’s tolerance and ICU length of stay.

### Data acquisition

After visual analysis, all subsequent analysis was performed using Matlab with the signal processing toolbox (Matlab 2012b, Natick, MA, USA). After detailed analysis of each recording, a single channel was selected for further analysis, known as C3/C4, placed centrally on the left and right hemisphere. This electrode location and configuration has been shown to be most representative in distinguishing between relevant sleep-states in healthy individuals with minimal EMG interference [23]. Single channel EEG has the added advantage of low complexity and, therefore, added speed of clinical setup for possible future application. The all-or-nothing functionality that comes with it reduces the risk of undetected electrode malfunction in future real-time analyses.

### Validation

The IDOS index was calculated for all outpatient and ICU recordings. Each day of ICU recording was treated as an individual recording during analysis. Although the purpose of the IDOS index is merely to display depth of sleep over time, and not to classify sleep into discrete stages, a semi-automatic comparison to R&K was performed to quantify face validity. To facilitate epoch-by-epoch comparison between the IDOS index and R&K classification, the index was averaged over 30-second segments, followed by manual threshold selection for the transition between sleep and wake with *a priori* knowledge of R&K classification for each entire day of recording. A single value of the IDOS index was selected that best resembled sleep onset and offset for each individual day of recording. The same was done for the transition from sleep to SWS. The resulting classifications into two (sleep and wake) and three (wake, non-SWS and SWS) classes were compared to R&K analysis. For purposes of meaningful comparison, the R&K classification was reduced to the same two and three classes by combining NREM1, NREM2 and REM to form non-SWS. SWS consisted of NREM3 and NREM4. Cohen’s Kappa statistic was used to evaluate agreement between both methods.

## Results

A total of 298.40 hours of control PSG recordings were obtained from 15 outpatients, with a mean (SD) recording time per patient of 19.89 (1.48) hours (Table 1). The average agreement for these recordings as defined by Cohen’s Kappa for three stage classification was 0.82 (0.06) (Table 2). For two stage classification, Kappa was slightly higher, at 0.84, with a SD of 0.08. The results of R&K analysis and IDOS index threshold selection for the 15 individual outpatient recordings can be found in the additional files [see Additional files 1, 2, 3, 4, 5, 6, 7, 8, 9, 10, 11, 12, 13, 14 and 15]. The corresponding agreement and sensitivity/specificity values for individual outpatient recordings can be found in an additional table [see Additional file 16].

**Table 1:** Patient characteristics for the control group and results of R&K analysis (number = 15)

**Characteristics**	**Control group, number = 15**
Male/Female, number	8/7
Age, mean (SD), years	42.9 (16.2)
BMI, mean (SD)	28.8 (9.3)
ESS^a^ score, mean (SD)	8.1 (4)
Average length of PSG, hours	19.9 (1.5)
TST^b^, mean (SD), hours:minutes	7:50 (1:02)
Sleep latency, means (SD), minutes	23 (10)
Time spent in each stage, mean (SD), % of TST^b^	
REM	21 (4)
S1	11 (6)
S2	46 (9)
S3	12 (8)
S4	10 (5)
Sleep efficiency^c^, mean (SD), %	93 (5)

^a^The Epworth Sleepiness Scale (ESS) is used to determine the level of daytime sleepiness. A score of 10 or more is considered sleepy, 18 or more is very sleepy. ^b^Total sleep time (TST) is the combined time spent in either sleep stage. ^c^Sleep efficiency is the ratio defined by the time spent asleep and the time spent in bed. BMI, body mass index; PSG, polysomnography; REM, rapid eye movement; R&K, Rechtschaffen and Kales; SD, standard deviation.

**Table 2:** Contingency table of the pooled results for outpatient recordings (number = 15)

		**IDOS**		
		**Wake**	**non-SWS**	**SWS**
	Wake	19,734	1,429	1
**R&K**	non-SWS	1,374	9,153	874
	SWS	19	653	2,571

The contingency table of visually scored polysomnographic classification (‘R&K’) versus the classification by IDOS index for the combined epochs of all 15 datasets is shown. The amount of the epochs classified as either wake, non-SWS or SWS are given for both methods. Rows represent the number of epochs scored as each state by conventional visual analysis according to R&K. Columns represent the classification of the same epochs by the IDOS index. The agreement for three classes was excellent (Cohen’s kappa coefficient = 0.82, SD = 0.06), agreement for two classes was slightly better (kappa = 0.84, SD = 0.09). IDOS, ICU Depth of Sleep; R&K, Rechtschaffen and Kales; SD, standard deviation; SWS, slow wave sleep.

The inclusion of five ICU patients yielded approximately nine days of PSG recording, or 205.38 hours. Patient characteristics are summarized in Table 3. Patient A was admitted to the ICU for plasmapheresis, as the main treatment for thrombotic thrombocytopenic purpura (TTP)/hemolytic uremic syndrome (HUS). Patient B was intubated and sedated with propofol on the third day of recording. Due to the dominant propofol induced EEG activity, this day of recording was excluded from further analysis. Patient C was included in this study while on mechanical ventilation, and was extubated on the second day. Patient D, with progressive neuromuscular disease, was admitted for non-invasive mechanical ventilation. Patient E was admitted for treatment of an arterial thrombus.

**Table 3:** ICU patient characteristics

**Patient**	**A**	**B**	**C**	**D**	**E**
Sex	F	F	M	M	F
BMI	25 to 30	20 to 25	30 to 35	20 to 25	>35
Age range, years	60 to 70	50 to 60	60 to 70	60 to 70	40 to 50
Days prior on ICU	2	2	9	10	2
Duration of inclusion, days	1.81	2.98	2.94	0.99	0.93
APACHE II^a^	14	35	-	17	9
APACHE IV^b^	58	98	45	54	30
TISS 76, mean^c^	15.5	32	19	8.5	17.6
Diagnosis on admittance	TTP	Bacterial pneumonia	Viral pneumonia	Bacterial pneumonia	Arterial thrombus
Mechanical ventilation, days	0	1	1	0	0
Length of ICU stay, days	3	10	13	30	5
Medication, per recording day, median Benzodiazepines, mg (Lorazepam eqv.)	0.3	0.5	0.3	0	1.8
Opioids, mg (morphine eqv.)	1.3	8	24.3	0	21.3
Propofol 2%, ml	0	38	0	0	0

^a^Acute Physiology and Chronic Health Evaluation (APACHE) II is a severity of illness scoring system, with scores ranging from 0 (best) to 71 (worst). All scores were determined using the most abnormal values in the first 24 hours of ICU admission. ^b^APACHE IV is a severity of illness scoring system, with scores ranging from 0 (best) to 150 (worst). All scores were determined using the most abnormal values in the first 24 hours of ICU admission. ^c^Therapeutic Intervention Scoring System (TISS 76) quantifies nursing workload into four classes depending on 76 points of therapeutic intervention. The mean is calculated from daily scores. BMI, body mass index; TTP, thrombotic thrombocytopenic purpura.

**Figure 3:**
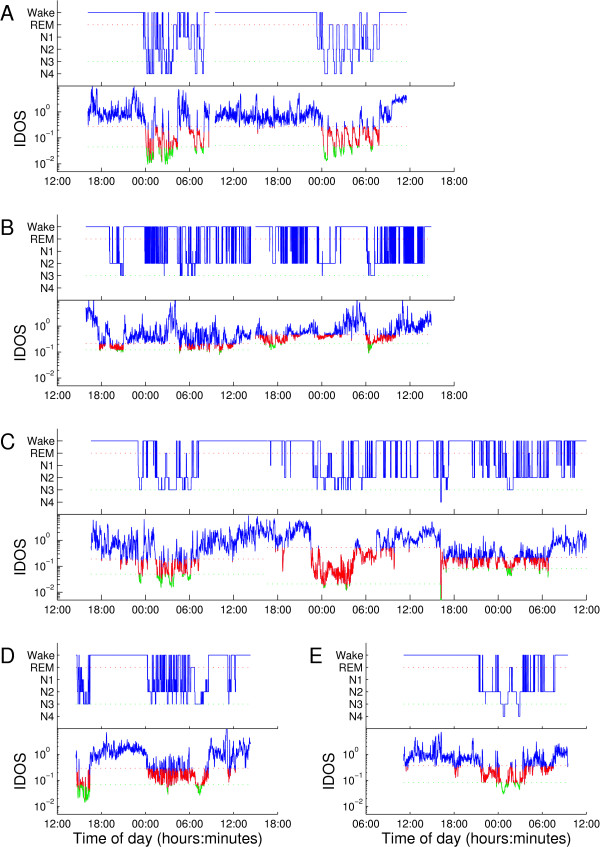
**Hypnogram after R&K classification of ICU patients with corresponding IDOS tracings.** The IDOS index, calculated from EEG channel C3/C4 is shown below the hypnograms after R&K analysis for all five ICU patients. Both R&K classification and the IDOS index of PSG recordings show relatively normal sleep for Patient **A**, particularly on the second night. The first night shows some arousals, but there is still transitioning to SWS. Nearly all sleep is seen during the night. Patient **B** shows severe fragmentation, with sleep spread evenly between day and night. In the hypnogram and IDOS tracing rapid switching between sleep and wake is visible. Patient **C**’s hypnogram shows a period of sleep in the first night, when the patient was non-sedated and mechanically ventilated. After extubation following the first night, sleep architecture seems to gradually deteriorate. Sleep on days two and three is fragmented with little SWS and no distinct sleep cycles in either tracing. Patient **D** shows severe fragmentation by awakenings and a period of daytime-sleep, visible in the hypnogram and IDOS tracing. Patient **E** shows a single ultradian sleep cycle around 2:00 in both tracings, followed by a period of fragmented sleep. All sleep occurred during the evening and night according to the hypnogram, although the IDOS tracing suggests short bursts of sleep during the day. EEG, electroencephalogram; IDOS, ICU Depth of Sleep; PSG, polysomnography; R&K, Rechtschaffen and Kales; SWS, slow wave sleep.

Per day manual threshold selection of the IDOS index resulted in similar amounts of the individual sleep stages (not shown) with a high variance of Cohen’s Kappa statistic between recordings. The agreement of both classification methods was excellent for patient A (Kappa = 0.90), reasonable for patient B (Kappa = 0.46), good for patient C (Kappa = 0.65), and excellent for patients D (Kappa = 0.83) and E (Kappa = 0.80). Average sensitivity (SD) was 0.88 (0.10) for wake epochs, 0.66 (0.15) for non-SWS, and 0.68 (0.22) for SWS. Average specificity (SD) was 0.87 (0.09) for wake, 0.69 (0.14) for sleep and 0.59 (0.27) for SWS. The classifications of ICU recordings by R&K and the IDOS index are explained in greater detail in Figure 3.

## Discussion

Using single channel EEG data, the IDOS index seems to be a promising and simple estimate of depth of sleep, even in the critically ill. Eliminating the need for human intervention in the analysis of the ICU acquired data results in fast, cost-effective and objective insight on ICU patients’ sleep. This opens up possibilities, not only for future large scale ICU sleep research, but also for the monitoring of individual ICU patients for targeted therapy to facilitate natural sleep. Calculating a simple ratio of gamma and delta frequency activity using only a single channel of EEG, however, has, to our knowledge, never been attempted as an index for depth of sleep.

So far, the study of sleep and wake in the ICU has relied heavily on PSG, a time-consuming and complex method of measuring several parameters most indicative of quality and quantity of sleep [1]. For most patients these recordings take place during the night; however, in the ICU, sleep is often not limited to the night alone and 24 hour PSG is preferred. The acquired data is manually scored in 30-second segments, which eventually amounts to large workloads and high costs. The R&K classification is for all intents and purposes relatively subjective and interrater agreement between individual scorers is consequently low in ICU recordings. Previous studies reporting interrater reliability for R&K analysis of ICU recordings show Kappa’s ranging from 0.56 to as low as 0.19 [24,25]. Using data from only a single EEG channel in a more promising and objective manner, similar results were achieved, without the specific need for other non-encephalographic signals. Although this method inherits some of the disadvantages of PSG, that is, the dependency on clean electrical activity in a high-tech environment and high variability of the EEG spectral composition of ICU patients, it simplifies and objectifies the practical aspects of depth of sleep measurement.

Attempting to categorize the EEG of sedated patients, or patients with significant neurologic disorders, into discrete stages of sleep seems ineffective. We, therefore, propose a more objective perspective on EEG activity, while still being capable of showing temporal changes in depth of sleep as they occur in ICU patients. To illustrate face validity with the current standard for depth of sleep, R&K analysis of PSG recordings, the IDOS index was manually classified into discrete stages. This method seems justifiable in outpatient recordings, where R&K is widely used. The comparison of IDOS to R&K in ICU patients is less obvious, however, since R&K interrater agreements are known to be low in these recordings. For future validation in a larger sample of critically ill patients, the correlation of the IDOS index with behavioral assessment, sedation scores, severity of illness and automated methods, such as SEF95 and BIS, needs to be determined.

The exact factors attributable to poor sleep quality in the ICU and their contributions in disturbing sleep are not yet known. Acute illness, patient-care interactions, light, pain, patient discomfort and noise are all factors that likely contribute to the frequent arousals and awakenings that ICU patients experience [1,8,9,13,26-28]. Also, sleep medication that is given to overcome these observed disturbances may result in a state that subjectively resembles sleep, but may not be as physically beneficial as true slow wave sleep [29]. To increase our understanding of the intricacies of sleep in the ICU, the effects of these potentially disturbing factors need to be studied closely in future studies. This requires application of new techniques, well suited for large scale application in the ICU, one of which could be the IDOS index.

The main disadvantage of this study was the limited number of recordings to investigate the practical considerations of the proposed technique in the ICU environment. Severity of illness scores varied significantly between patients, as did other patient characteristics such as days prior on ICU and administered doses of benzodiazepines and opioids. Although results do not justify immediate use in ICU sleep research, they do warrant further development and testing in a more representative cross-section of the general ICU population.

From a technical standpoint several limitations of the study should be mentioned. One of the main technical disadvantages of spectral analysis of EEG data is the large inter-individual difference between recordings and patients. Despite attempts to minimize these differences by filtering and normalization, there are still visible changes from one day of recording to the next for the same ICU patient and also between individual patients. Thresholds between days varied in the ICU recordings, and thresholds between patients varied significantly in both groups. This made classification difficult in the most abnormal recordings in the ICU, and hampered agreement with R&K, but does not necessarily diminish the value as an indicator for depth of sleep.

The choice to involve gamma-band electrical activity in the analysis of sleep is controversial. The possibility that EMG is responsible for the majority of electrical activity in this range does, however, not negate the fact that it is usable as a variable for sleep state analysis in most non-sedated patients. More importantly, potential noise or artefacts from nursing care activities or other sources could be most apparent in the gamma-band and, therefore, heavily skew the IDOS index. Minimizing the effects of these changes on the parameters used to determine depth of sleep has first priority in further development of this technique.

The study of sleep in the ICU is a growing field of interest. Patients barely seem to sleep for prolonged periods of time, if at all, and all criteria for the diagnosis of delirium may be caused by loss of sleep [1]. Our perception of sleep and its relevance in ICU patients’ wellbeing has changed thanks to the introduction of small scale ICU sleep research relying heavily on PSG. Before large scale interventional studies can be undertaken effectively and efficiently, unsupervised, simple, robust and preferably real-time analysis of sleep is needed. With the IDOS index the first step has been made, using only simple existing techniques, towards scalable ICU depth of sleep monitoring.

## Conclusions

Our IDOS index showed excellent agreement with traditional R&K analysis of recordings exhibiting normal sleep, for two and three stage classifications. This indicates solid performance of the index in measuring depth of sleep. The high face-validity in the control group is also reflected in ICU patients with relatively normal sleep. Although agreement between both methods was highly variable in ICU patients, the average agreement seems promising for future clinical and research application. Overall we conclude that using the new IDOS index, depth of sleep can be determined reliably using only single channel EEG data from outpatient recordings. Future efforts will focus on validating and fine-tuning the index to be used in large scale ICU sleep research.

## Key messages

• We introduce a novel index, based on physiologically relevant EEG features, that allows monitoring of depth of sleep using a single EEG channel, designed specifically for the study of sleep in the ICU.

• Our index showed remarkable resemblance to Rechtschaffen & Kales’ method of EEG sleep classification, with excellent agreement in recordings of normal sleep.

• In five ICU patient recordings the agreement was lower, but comparable to interrater agreement of R&K classification.

## Abbreviations

APACHE: Acute Physiology and Chronic Health Evaluation; BMI: body mass index; ECG: electrocardiography; EEG: electroencephalography; EMG: electromyography; EOG: electro-oculography; ESS: Epworth Sleepiness Scale; HUS: hemolytic uremic syndrome; PSD: power spectral density; PSG: polysomnography; R&K: Rechtschaffen and Kales; REM: rapid eye movement; SD: standard deviation; SWS: slow wave sleep; TSS: therapeutic intervention scoring system; TST: total sleep time; TTP: thrombotic thrombocytopenic purpura.

## Competing interests

The authors declare that they have no competing interests.

## Authors’ contributions

LR conceived the ICU measurements, enrolled patients, collated the data, analyzed and interpreted results, conceived and undertook the literature review, and drafted the manuscript. JvdH analyzed PSG recordings of ICU and polyclinic patients, contributed to the design and execution of the EEG measurements, contributed substantially to the concept of the IDOS index, and revised the manuscript. MvP provided methodological advice regarding EEG monitoring and interpretation of results, participated in design of the study, analyzed results of the IDOS index, and significantly revised the manuscript. JT performed the initial literature review, designed the protocol of the clinical study, organized clinical measurements, assisted in inclusion of ICU patients, and drafted the manuscript. WD provided methodological and organizational advice and assistance in inclusion of ICU patients, interpreted results and contributed substantially to the first draft of the manuscript. All authors read, and approved the final manuscript.

## Supplementary Material

Additional file 1 **Hypnogram and IDOS index of outpatient recording 1.** The hypnogram resulting from R&K analysis (figure A) of the outpatient recording is shown. The IDOS index for the same recording. The IDOS index (figure B) of the same recording shows similar transitions of depth of sleep. Colors indicate the separation into three classes; wake (blue), non-SWS (red) and SWS (green). The transition from wake to sleep (red dotted line) and from non-SWS to SWS (green dotted line) are also given. The same colors for sleep stages are used for both figures. IDOS, ICU Depth of Sleep; R&K, Rechtschaffen and Kales; SWS, slow wave sleep. Click here for file

Additional file 2 **Hypnogram and IDOS index of outpatient recording 2.** The hypnogram resulting from R&K analysis (figure A) of the outpatient recording is shown. The IDOS index for the same recording. The IDOS index (figure B) of the same recording shows similar transitions of depth of sleep. Colors indicate the separation into three classes: wake (blue), non-SWS (red) and SWS (green). The transition from wake to sleep (red dotted line) and from non-SWS to SWS (green dotted line) are also given. The same colors for sleep stages are used for both figures. IDOS, ICU Depth of Sleep; R&K, Rechtschaffen and Kales; SWS, slow wave sleep. Click here for file

Additional file 3 **Hypnogram and IDOS index of outpatient recording 3. **The hypnogram resulting from R&K analysis (figure A) of the outpatient recording is shown. The IDOS index for the same recording. The IDOS index (figure B) of the same recording shows similar transitions of depth of sleep. Colors indicate the separation into three classes: wake (blue), non-SWS (red) and SWS (green). The transition from wake to sleep (red dotted line) and from non-SWS to SWS (green dotted line) are also given. The same colors for sleep stages are used for both figures. IDOS, ICU Depth of Sleep; R&K, Rechtschaffen and Kales; SWS, slow wave sleep. Click here for file

Additional file 4 **Hypnogram and IDOS index of outpatient recording 4.** The hypnogram resulting from R&K analysis (figure A) of the outpatient recording is shown. The IDOS index for the same recording. The IDOS index (figure B) of the same recording shows similar transitions of depth of sleep. Colors indicate the separation into three classes: wake (blue), non-SWS (red) and SWS (green). The transition from wake to sleep (red dotted line) and from non-SWS to SWS (green dotted line) are also given. The same colors for sleep stages are used for both figures. IDOS, ICU Depth of Sleep; R&K, Rechtschaffen and Kales; SWS, slow wave sleep. Click here for file

Additional file 5 **Hypnogram and IDOS index of outpatient recording 5.** The hypnogram resulting from R&K analysis (figure A) of the outpatient recording is shown. The IDOS index for the same recording. The IDOS index (figure B) of the same recording shows similar transitions of depth of sleep. Colors indicate the separation into three classes: wake (blue), non-SWS (red) and SWS (green). The transition from wake to sleep (red dotted line) and from non-SWS to SWS (green dotted line) are also given. The same colors for sleep stages are used for both figures. IDOS, ICU Depth of Sleep; R&K, Rechtschaffen and Kales; SWS, slow wave sleep. Click here for file

Additional file 6 **Hypnogram and IDOS index of outpatient recording 6.** The hypnogram resulting from R&K analysis (figure A) of the outpatient recording is shown. The IDOS index for the same recording. The IDOS index (figure B) of the same recording shows similar transitions of depth of sleep. Colors indicate the separation into three classes: wake (blue), non-SWS (red) and SWS (green). The transition from wake to sleep (red dotted line) and from non-SWS to SWS (green dotted line) are also given. The same colors for sleep stages are used for both figures. IDOS, ICU Depth of Sleep; R&K, Rechtschaffen and Kales; SWS, slow wave sleep. Click here for file

Additional file 7 **Hypnogram and IDOS index of outpatient recording 7.** The hypnogram resulting from R&K analysis (figure A) of the outpatient recording is shown. The IDOS index for the same recording. The IDOS index (figure B) of the same recording shows similar transitions of depth of sleep. Colors indicate the separation into three classes: wake (blue), non-SWS (red) and SWS (green). The transition from wake to sleep (red dotted line) and from non-SWS to SWS (green dotted line) are also given. The same colors for sleep stages are used for both figures. IDOS, ICU Depth of Sleep; R&K, Rechtschaffen and Kales; SWS, slow wave sleep. Click here for file

Additional file 8 **Hypnogram and IDOS index of outpatient recording 8.** The hypnogram resulting from R&K analysis (figure A) of the outpatient recording is shown. The IDOS index for the same recording. The IDOS index (figure B) of the same recording shows similar transitions of depth of sleep. Colors indicate the separation into three classes: wake (blue), non-SWS (red) and SWS (green). The transition from wake to sleep (red dotted line) and from non-SWS to SWS (green dotted line) are also given. The same colors for sleep stages are used for both figures. IDOS, ICU Depth of Sleep; R&K, Rechtschaffen and Kales; SWS, slow wave sleep. Click here for file

Additional file 9 **Hypnogram and IDOS index of outpatient recording 9.** The hypnogram resulting from R&K analysis (figure A) of the outpatient recording is shown. The IDOS index for the same recording. The IDOS index (figure B) of the same recording shows similar transitions of depth of sleep. Colors indicate the separation into three classes: wake (blue), non-SWS (red) and SWS (green). The transition from wake to sleep (red dotted line) and from non-SWS to SWS (green dotted line) are also given. The same colors for sleep stages are used for both figures. IDOS, ICU Depth of Sleep; R&K, Rechtschaffen and Kales; SWS, slow wave sleep. Click here for file

Additional file 10 **Hypnogram and IDOS index of outpatient recording 10.** The hypnogram resulting from R&K analysis (figure A) of the outpatient recording is shown. The IDOS index for the same recording. The IDOS index (figure B) of the same recording shows similar transitions of depth of sleep. Colors indicate the separation into three classes: wake (blue), non-SWS (red) and SWS (green). The transition from wake to sleep (red dotted line) and from non-SWS to SWS (green dotted line) are also given. The same colors for sleep stages are used for both figures. IDOS, ICU Depth of Sleep; R&K, Rechtschaffen and Kales; SWS, slow wave sleep. Click here for file

Additional file 11 **Hypnogram and IDOS index of outpatient recording 11.** The hypnogram resulting from R&K analysis (figure A) of the outpatient recording is shown. The IDOS index for the same recording. The IDOS index (figure B) of the same recording shows similar transitions of depth of sleep. Colors indicate the separation into three classes: wake (blue), non-SWS (red) and SWS (green). The transition from wake to sleep (red dotted line) and from non-SWS to SWS (green dotted line) are also given. The same colors for sleep stages are used for both figures. IDOS, ICU Depth of Sleep; R&K, Rechtschaffen and Kales; SWS, slow wave sleep. Click here for file

Additional file 12 **Hypnogram and IDOS index of outpatient recording 12.** The hypnogram resulting from R&K analysis (figure A) of the outpatient recording is shown. The IDOS index for the same recording. The IDOS index (figure B) of the same recording shows similar transitions of depth of sleep. Colors indicate the separation into three classes: wake (blue), non-SWS (red) and SWS (green). The transition from wake to sleep (red dotted line) and from non-SWS to SWS (green dotted line) are also given. The same colors for sleep stages are used for both figures. IDOS, ICU Depth of Sleep; R&K, Rechtschaffen and Kales; SWS, slow wave sleep. Click here for file

Additional file 13 **Hypnogram and IDOS index of outpatient recording 13.** The hypnogram resulting from R&K analysis (figure A) of the outpatient recording is shown. The IDOS index for the same recording. The IDOS index (figure B) of the same recording shows similar transitions of depth of sleep. Colors indicate the separation into three classes: wake (blue), non-SWS (red) and SWS (green). The transition from wake to sleep (red dotted line) and from non-SWS to SWS (green dotted line) are also given. The same colors for sleep stages are used for both figures. IDOS, ICU Depth of Sleep; R&K, Rechtschaffen and Kales; SWS, slow wave sleep. Click here for file

Additional file 14 **Hypnogram and IDOS index of outpatient recording 14.** The hypnogram resulting from R&K analysis (figure A) of the outpatient recording is shown. The IDOS index for the same recording. The IDOS index (figure B) of the same recording shows similar transitions of depth of sleep. Colors indicate the separation into three classes: wake (blue), non-SWS (red) and SWS (green). The transition from wake to sleep (red dotted line) and from non-SWS to SWS (green dotted line) are also given. The same colors for sleep stages are used for both figures. IDOS, ICU Depth of Sleep; R&K, Rechtschaffen and Kales; SWS, slow wave sleep. Click here for file

Additional file 15 **Hypnogram and IDOS index of outpatient recording 15.** The hypnogram resulting from R&K analysis (figure A) of the outpatient recording is shown. The IDOS index for the same recording. The IDOS index (figure B) of the same recording shows similar transitions of depth of sleep. Colors indicate the separation into three classes: wake (blue), non-SWS (red) and SWS (green). The transition from wake to sleep (red dotted line) and from non-SWS to SWS (green dotted line) are also given. The same colors for sleep stages are used for both figures. IDOS, ICU Depth of Sleep; R&K, Rechtschaffen and Kales; SWS, slow wave sleep. Click here for file

Additional file 16 **Table S4 Agreement and sensitivity/specificity of threshold selection of IDOS index (n = 15).** Classification by IDOS index is compared to R&K analysis for individual recordings of normal sleep in outpatient recordings. IDOS, ICU Depth of Sleep; R&K, Rechtschaffen and Kales. Click here for file
