# Supplementary figures and images for: Intensive care unit depth of sleep: proof of concept of a simple electroencephalography index in the non-sedated
Source: Crit Care. 2014 Apr 9;18(2):R66. doi: 10.1186/cc13823 (PMC4057034; doi:10.1186/cc13823)

A

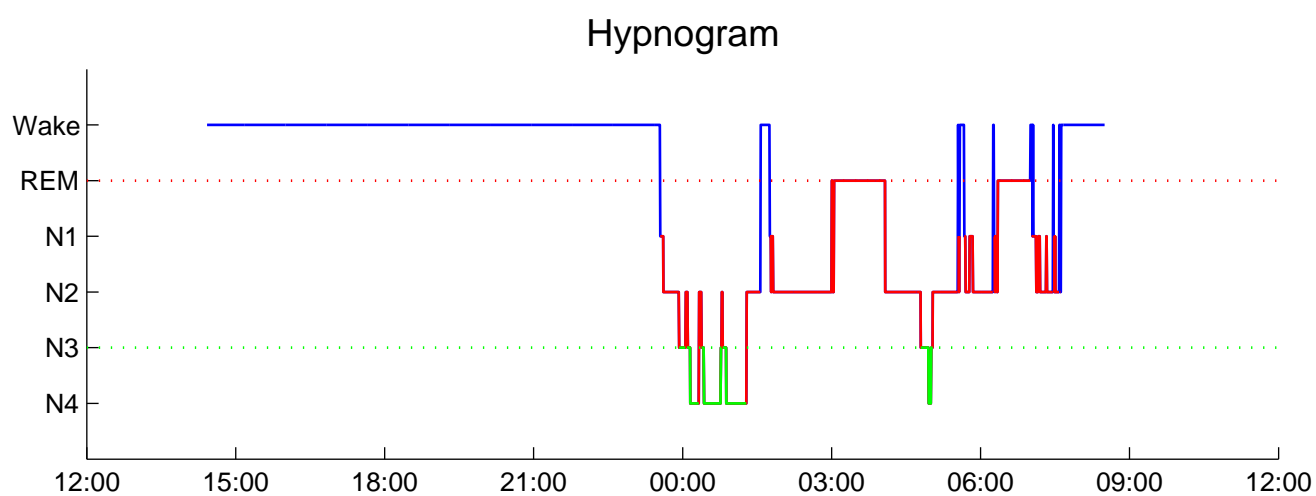

B

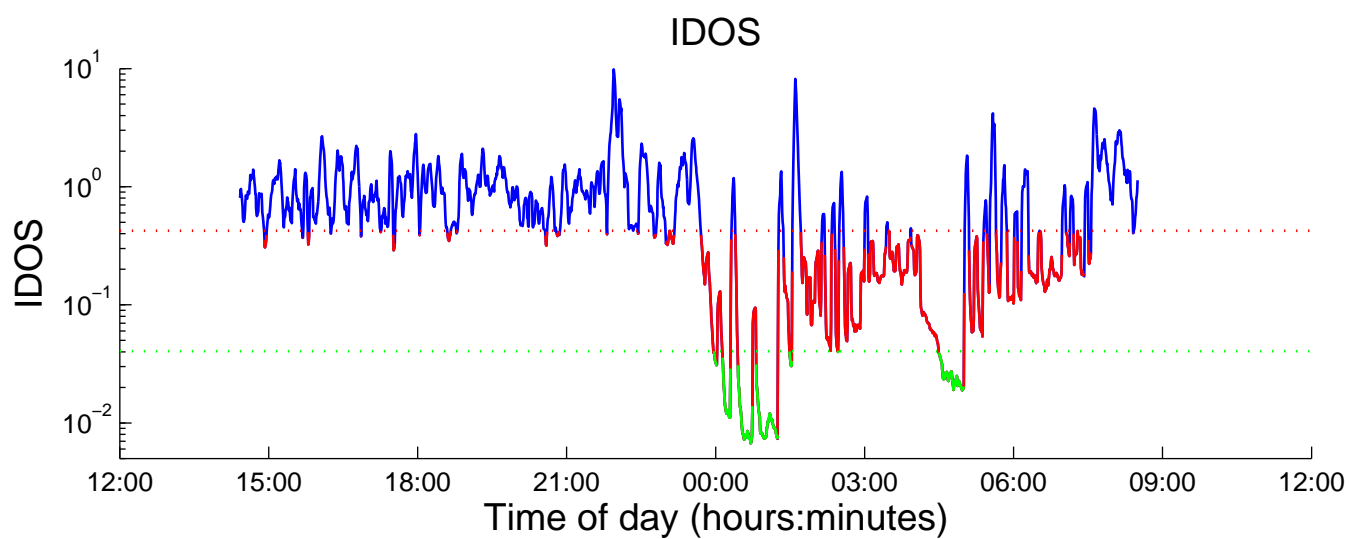

Supplement: Additional file 1 — Hypnogram and IDOS index of outpatient recording 1. The hypnogram resulting from R&K analysis (figure A) of the outpatient recording is shown. The IDOS index for the same recording. The IDOS index (figure B) of the same recording shows similar transitions of depth of sleep. Colors indicate the separation into three classes; wake (blue), non-SWS (red) and SWS (green). The transition from wake to sleep (red dotted line) and from non-SWS to SWS (green dotted line) are also given. The same colors for sleep stages are used for both figures. IDOS, ICU Depth of Sleep; R&K, Rechtschaffen and Kales; SWS, slow wave sleep. [file cc13823-S1.pdf]

A

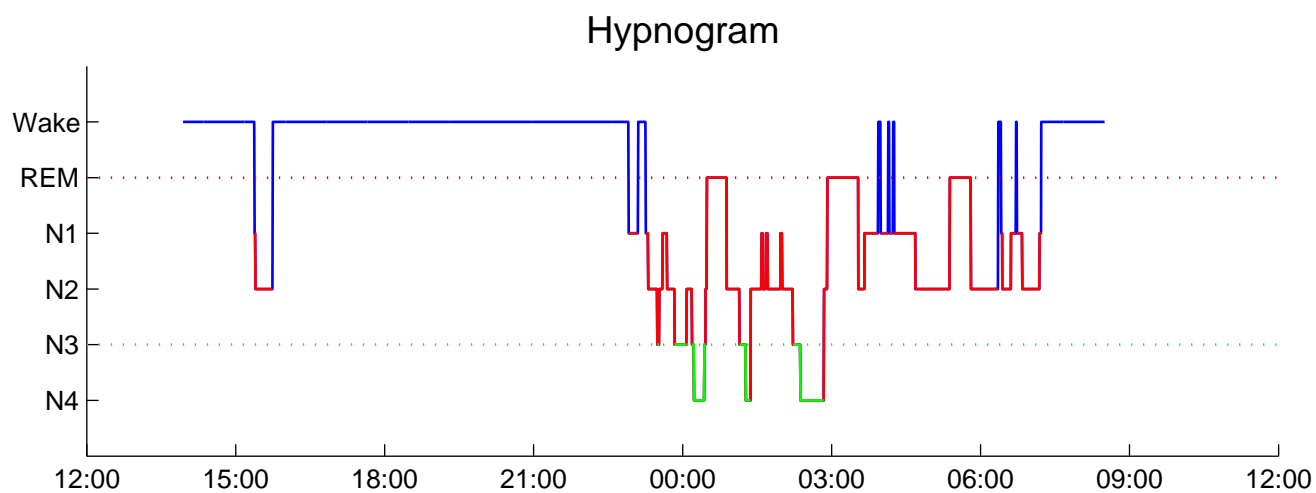

# B

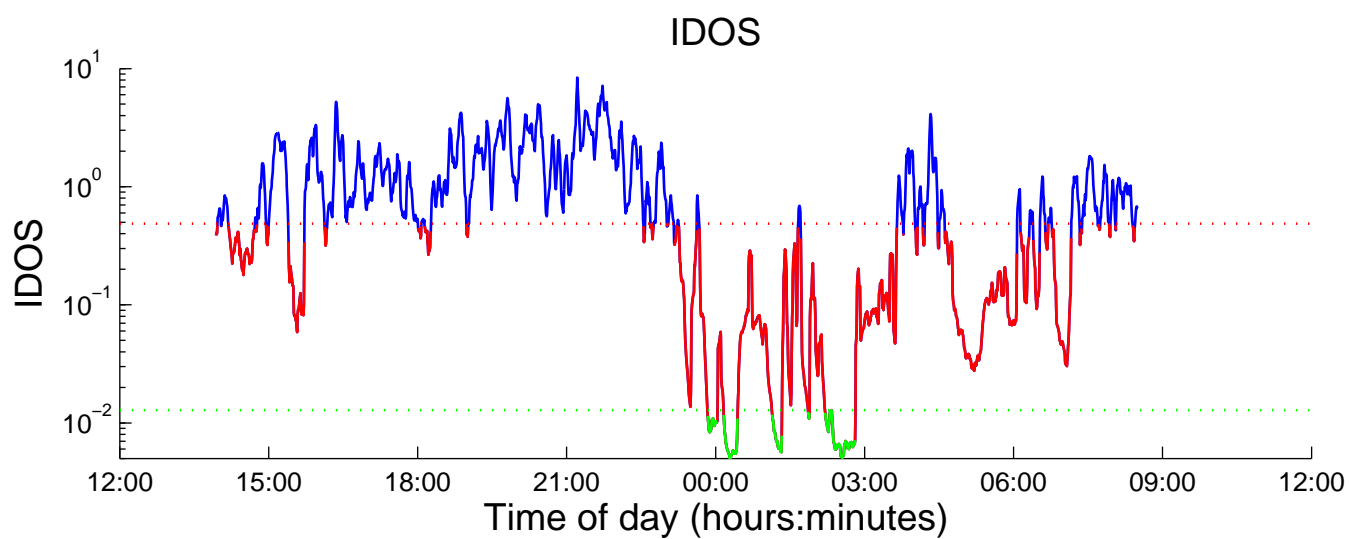

Supplement: Additional file 2 — Hypnogram and IDOS index of outpatient recording 2. The hypnogram resulting from R&K analysis (figure A) of the outpatient recording is shown. The IDOS index for the same recording. The IDOS index (figure B) of the same recording shows similar transitions of depth of sleep. Colors indicate the separation into three classes: wake (blue), non-SWS (red) and SWS (green). The transition from wake to sleep (red dotted line) and from non-SWS to SWS (green dotted line) are also given. The same colors for sleep stages are used for both figures. IDOS, ICU Depth of Sleep; R&K, Rechtschaffen and Kales; SWS, slow wave sleep. [file cc13823-S2.pdf]

A

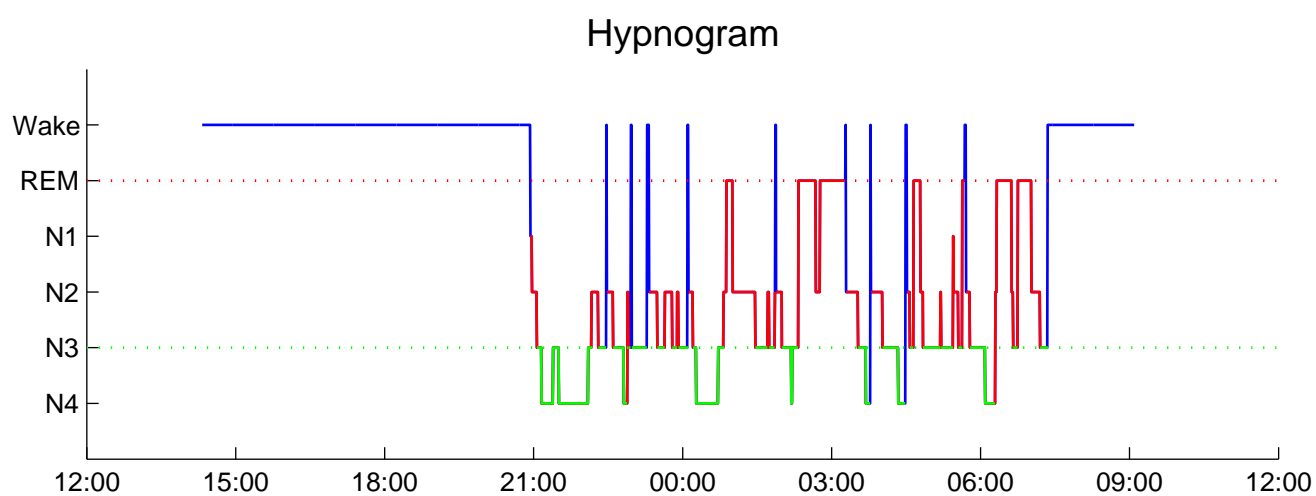

B

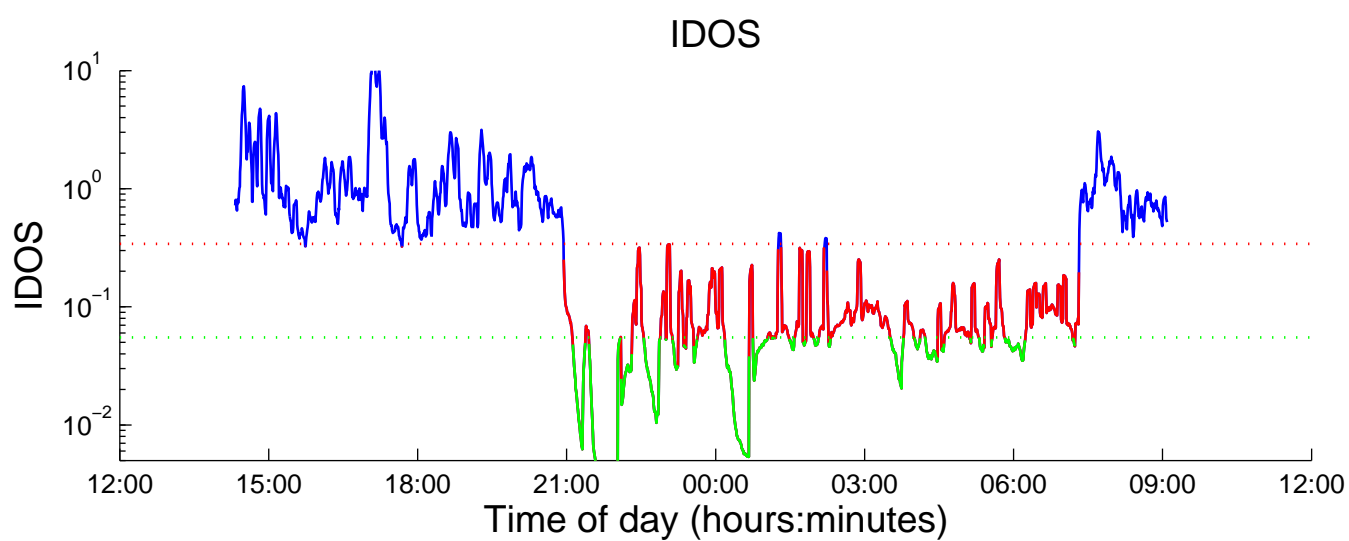

Supplement: Additional file 3 — Hypnogram and IDOS index of outpatient recording 3. The hypnogram resulting from R&K analysis (figure A) of the outpatient recording is shown. The IDOS index for the same recording. The IDOS index (figure B) of the same recording shows similar transitions of depth of sleep. Colors indicate the separation into three classes: wake (blue), non-SWS (red) and SWS (green). The transition from wake to sleep (red dotted line) and from non-SWS to SWS (green dotted line) are also given. The same colors for sleep stages are used for both figures. IDOS, ICU Depth of Sleep; R&K, Rechtschaffen and Kales; SWS, slow wave sleep. [file cc13823-S3.pdf]

A

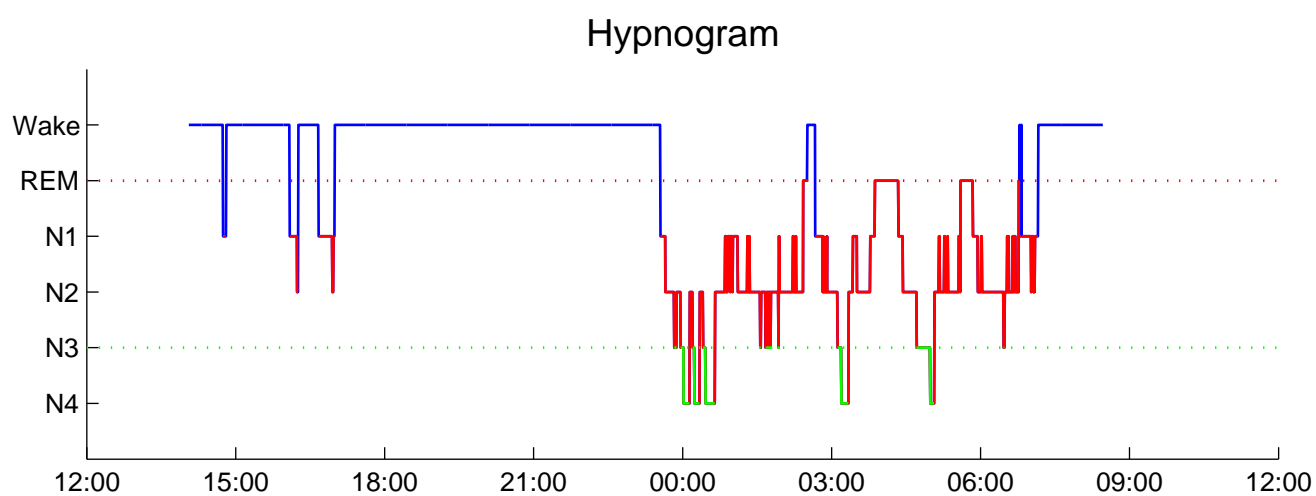

B

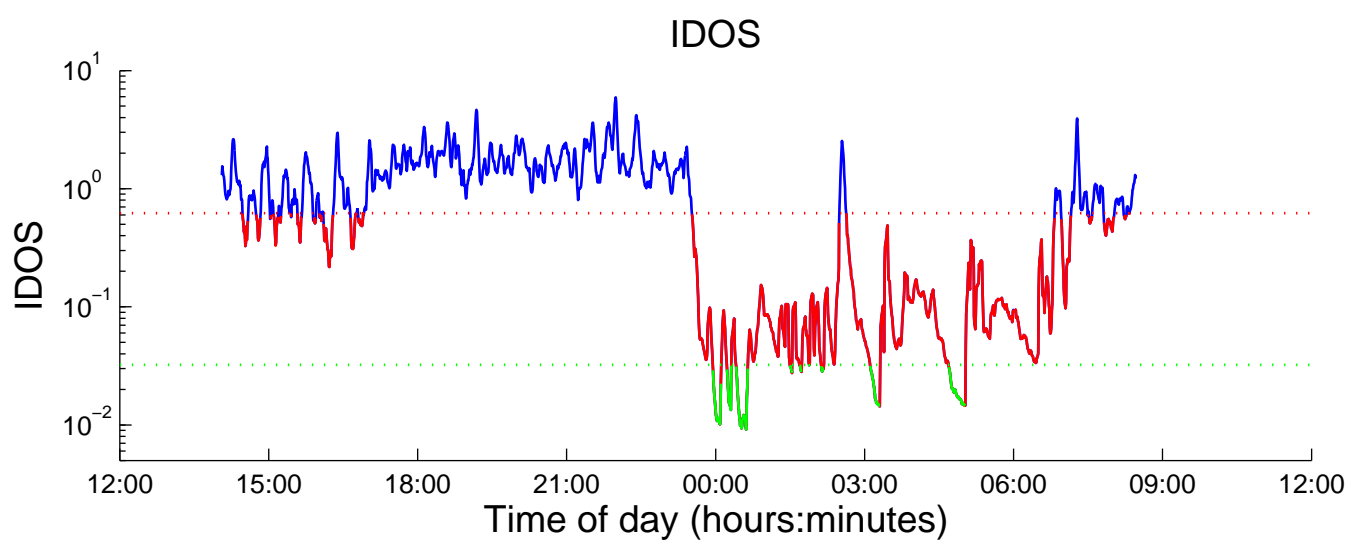

Supplement: Additional file 4 — Hypnogram and IDOS index of outpatient recording 4. The hypnogram resulting from R&K analysis (figure A) of the outpatient recording is shown. The IDOS index for the same recording. The IDOS index (figure B) of the same recording shows similar transitions of depth of sleep. Colors indicate the separation into three classes: wake (blue), non-SWS (red) and SWS (green). The transition from wake to sleep (red dotted line) and from non-SWS to SWS (green dotted line) are also given. The same colors for sleep stages are used for both figures. IDOS, ICU Depth of Sleep; R&K, Rechtschaffen and Kales; SWS, slow wave sleep. [file cc13823-S4.pdf]

A

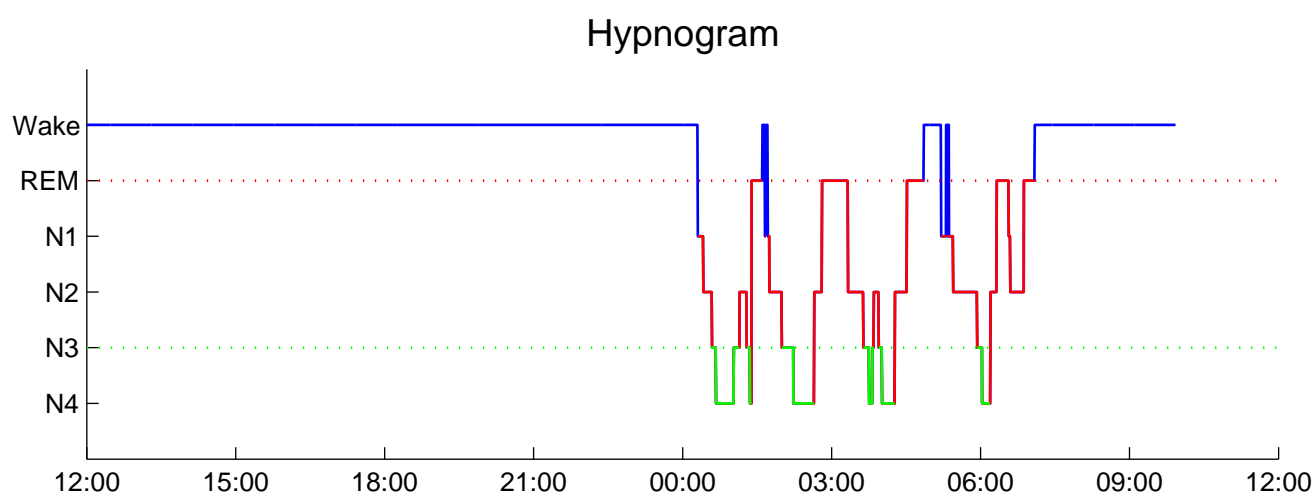

B

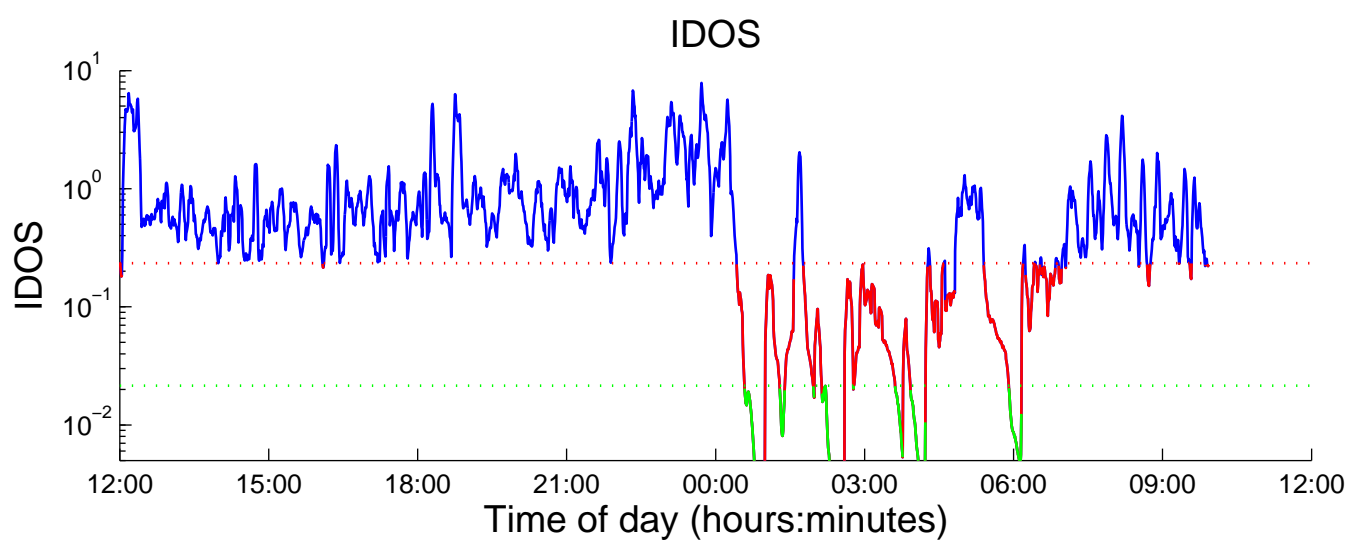

Supplement: Additional file 5 — Hypnogram and IDOS index of outpatient recording 5. The hypnogram resulting from R&K analysis (figure A) of the outpatient recording is shown. The IDOS index for the same recording. The IDOS index (figure B) of the same recording shows similar transitions of depth of sleep. Colors indicate the separation into three classes: wake (blue), non-SWS (red) and SWS (green). The transition from wake to sleep (red dotted line) and from non-SWS to SWS (green dotted line) are also given. The same colors for sleep stages are used for both figures. IDOS, ICU Depth of Sleep; R&K, Rechtschaffen and Kales; SWS, slow wave sleep. [file cc13823-S5.pdf]

A

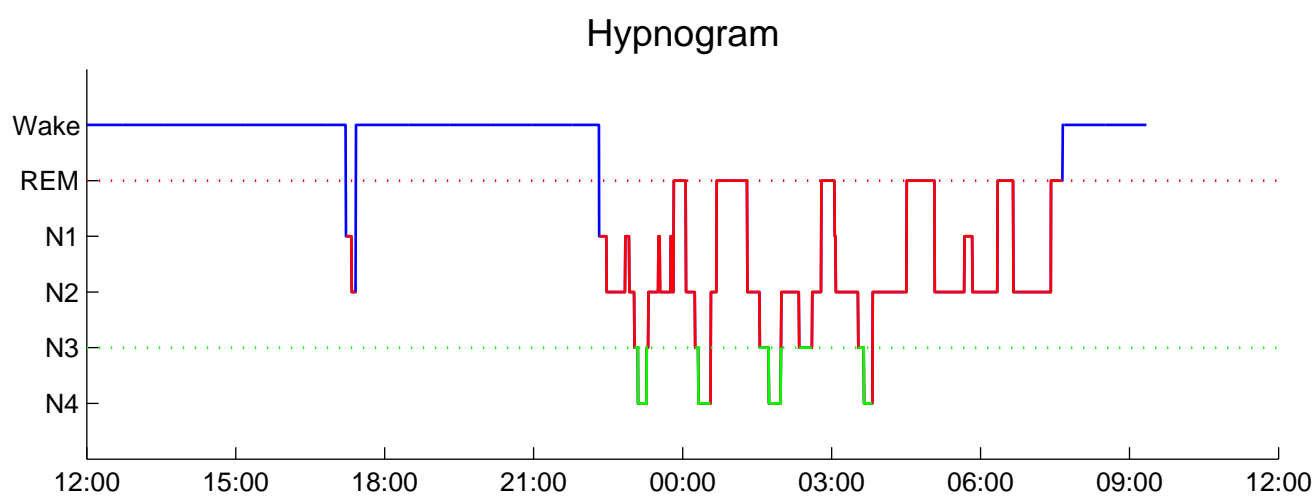

B

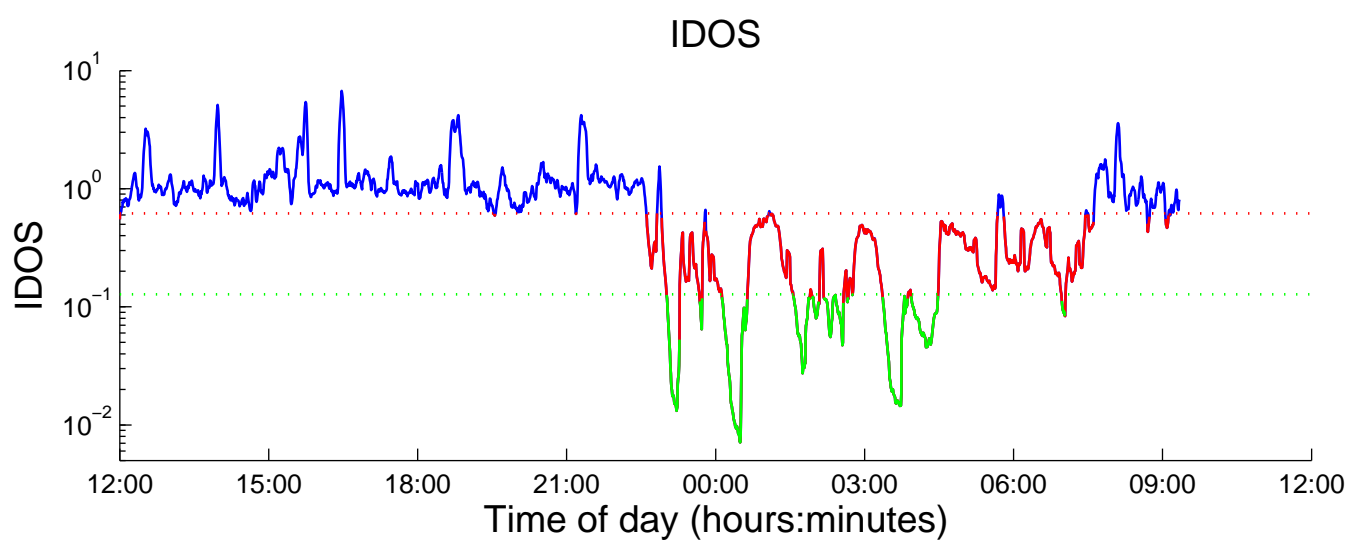

Supplement: Additional file 6 — Hypnogram and IDOS index of outpatient recording 6. The hypnogram resulting from R&K analysis (figure A) of the outpatient recording is shown. The IDOS index for the same recording. The IDOS index (figure B) of the same recording shows similar transitions of depth of sleep. Colors indicate the separation into three classes: wake (blue), non-SWS (red) and SWS (green). The transition from wake to sleep (red dotted line) and from non-SWS to SWS (green dotted line) are also given. The same colors for sleep stages are used for both figures. IDOS, ICU Depth of Sleep; R&K, Rechtschaffen and Kales; SWS, slow wave sleep. [file cc13823-S6.pdf]

A

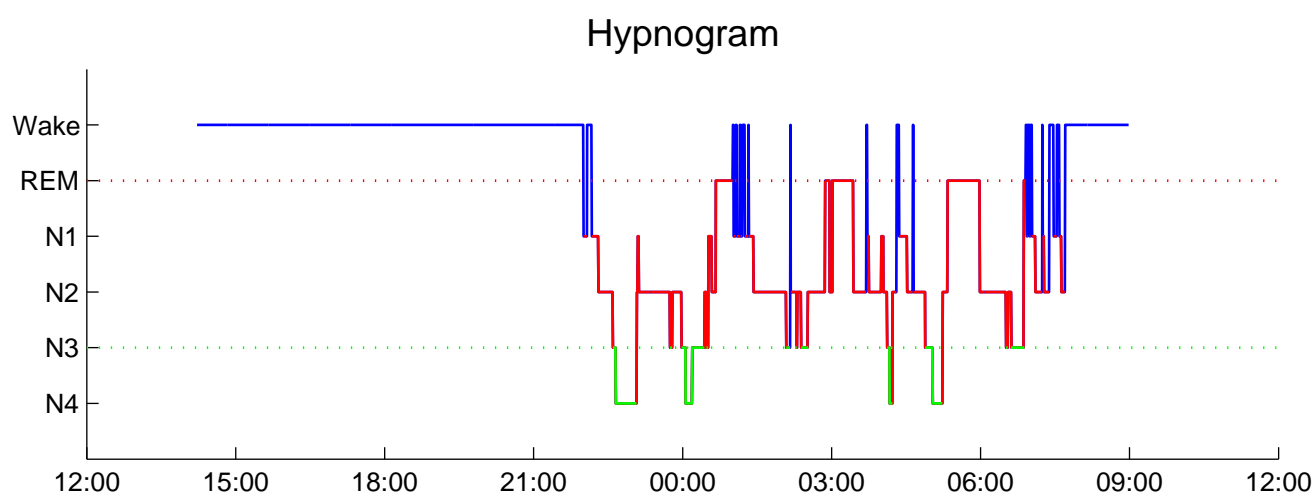

B

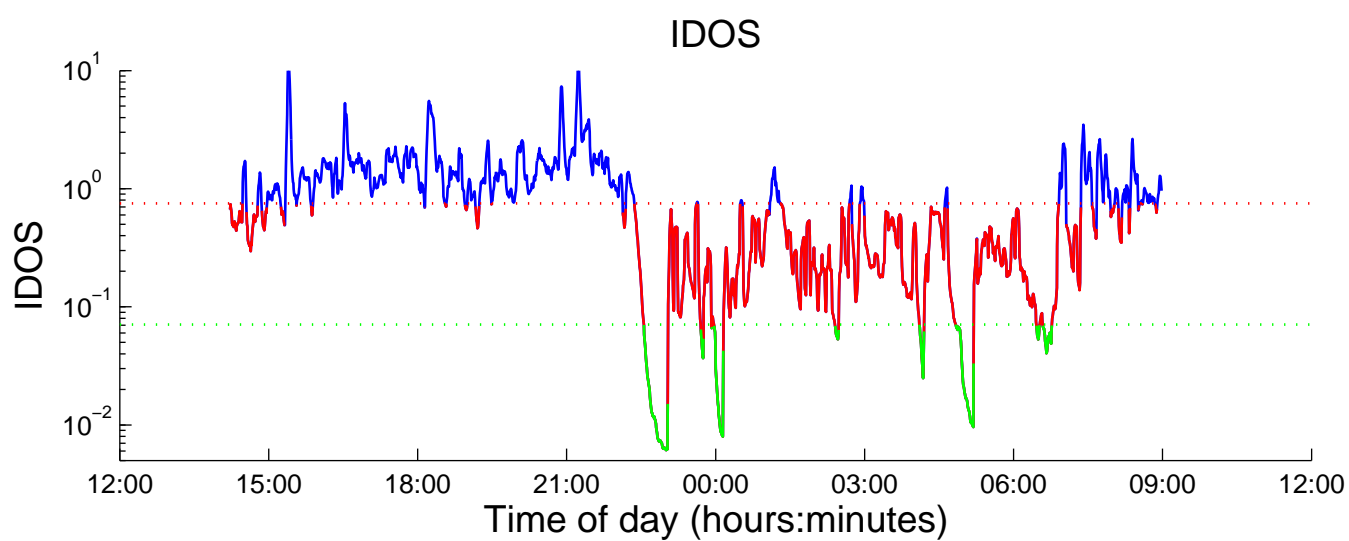

Supplement: Additional file 7 — Hypnogram and IDOS index of outpatient recording 7. The hypnogram resulting from R&K analysis (figure A) of the outpatient recording is shown. The IDOS index for the same recording. The IDOS index (figure B) of the same recording shows similar transitions of depth of sleep. Colors indicate the separation into three classes: wake (blue), non-SWS (red) and SWS (green). The transition from wake to sleep (red dotted line) and from non-SWS to SWS (green dotted line) are also given. The same colors for sleep stages are used for both figures. IDOS, ICU Depth of Sleep; R&K, Rechtschaffen and Kales; SWS, slow wave sleep. [file cc13823-S7.pdf]

A

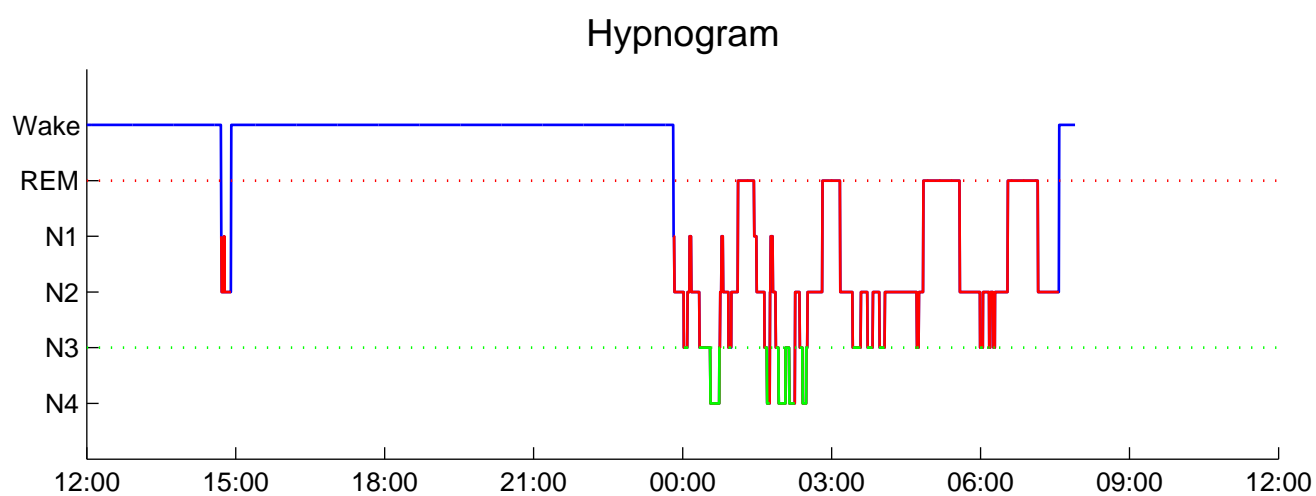

B

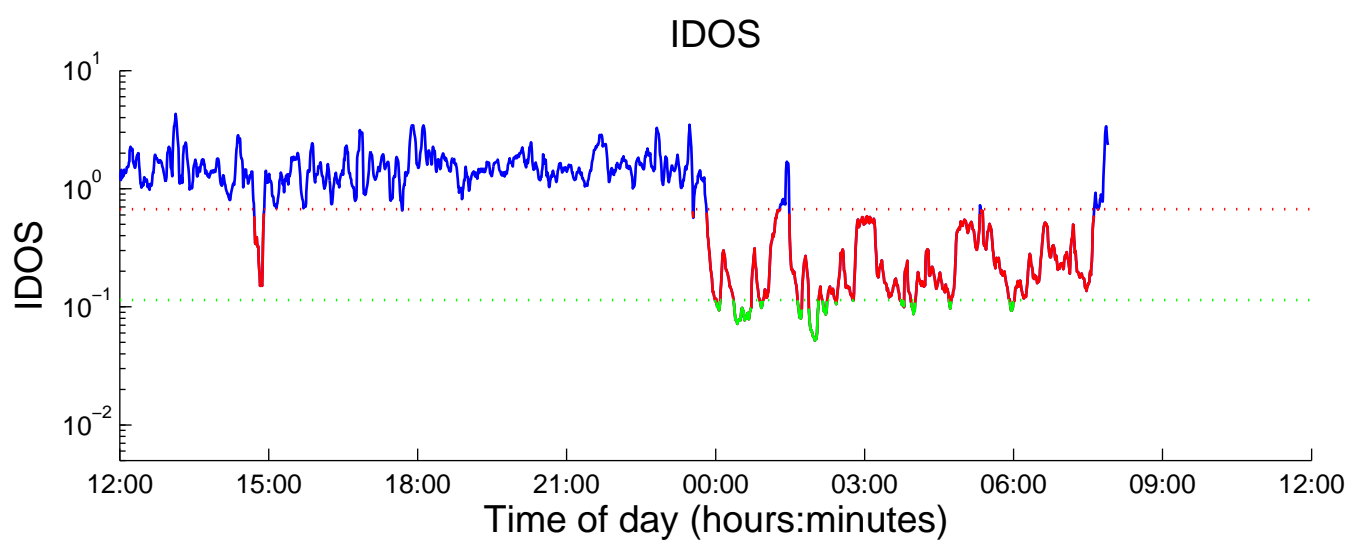

Supplement: Additional file 8 — Hypnogram and IDOS index of outpatient recording 8. The hypnogram resulting from R&K analysis (figure A) of the outpatient recording is shown. The IDOS index for the same recording. The IDOS index (figure B) of the same recording shows similar transitions of depth of sleep. Colors indicate the separation into three classes: wake (blue), non-SWS (red) and SWS (green). The transition from wake to sleep (red dotted line) and from non-SWS to SWS (green dotted line) are also given. The same colors for sleep stages are used for both figures. IDOS, ICU Depth of Sleep; R&K, Rechtschaffen and Kales; SWS, slow wave sleep. [file cc13823-S8.pdf]

# A

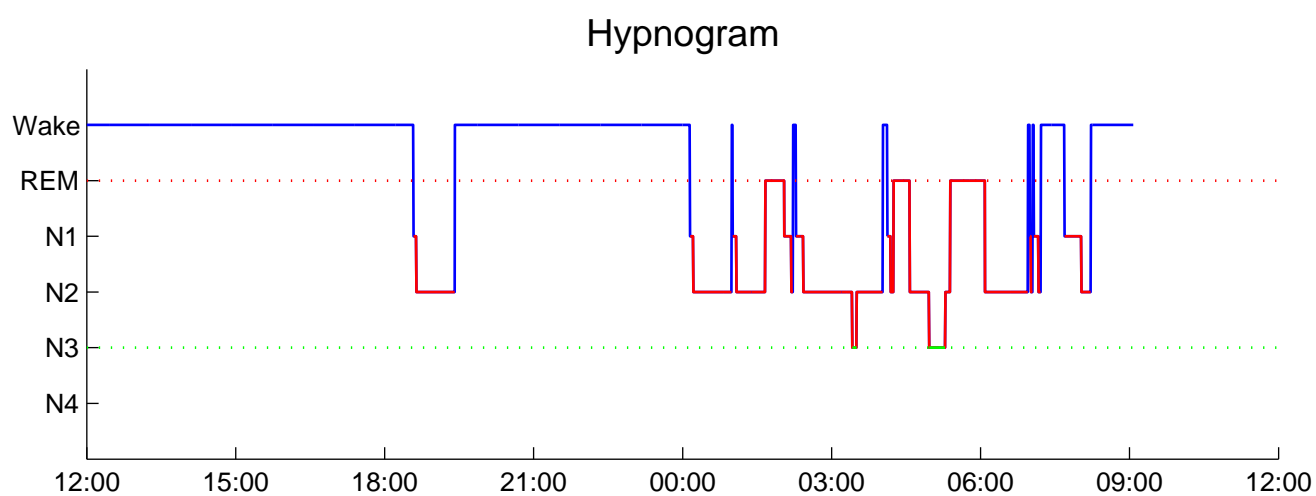

# B

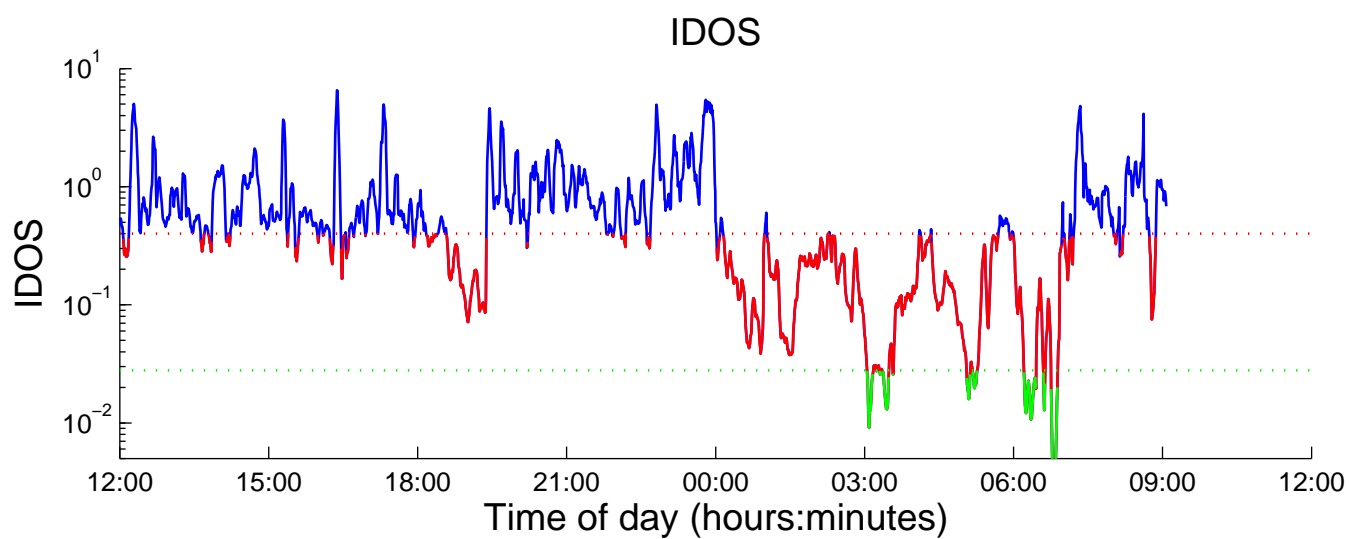

Supplement: Additional file 9 — Hypnogram and IDOS index of outpatient recording 9. The hypnogram resulting from R&K analysis (figure A) of the outpatient recording is shown. The IDOS index for the same recording. The IDOS index (figure B) of the same recording shows similar transitions of depth of sleep. Colors indicate the separation into three classes: wake (blue), non-SWS (red) and SWS (green). The transition from wake to sleep (red dotted line) and from non-SWS to SWS (green dotted line) are also given. The same colors for sleep stages are used for both figures. IDOS, ICU Depth of Sleep; R&K, Rechtschaffen and Kales; SWS, slow wave sleep. [file cc13823-S9.pdf]

A

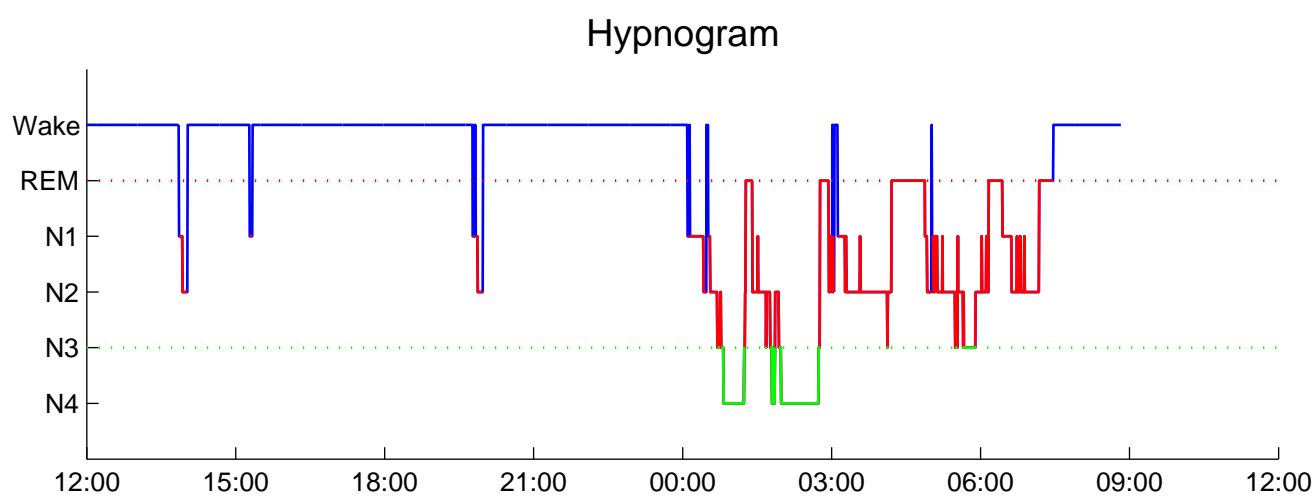

B

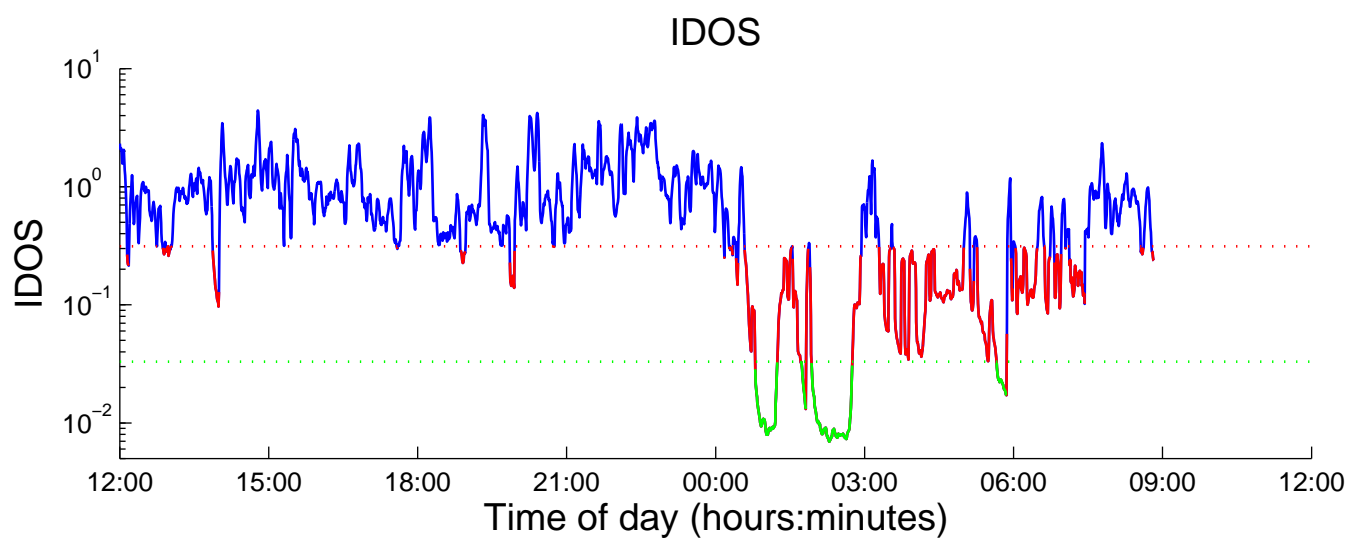

Supplement: Additional file 10 — Hypnogram and IDOS index of outpatient recording 10. The hypnogram resulting from R&K analysis (figure A) of the outpatient recording is shown. The IDOS index for the same recording. The IDOS index (figure B) of the same recording shows similar transitions of depth of sleep. Colors indicate the separation into three classes: wake (blue), non-SWS (red) and SWS (green). The transition from wake to sleep (red dotted line) and from non-SWS to SWS (green dotted line) are also given. The same colors for sleep stages are used for both figures. IDOS, ICU Depth of Sleep; R&K, Rechtschaffen and Kales; SWS, slow wave sleep. [file cc13823-S10.pdf]

A

Hypnogram

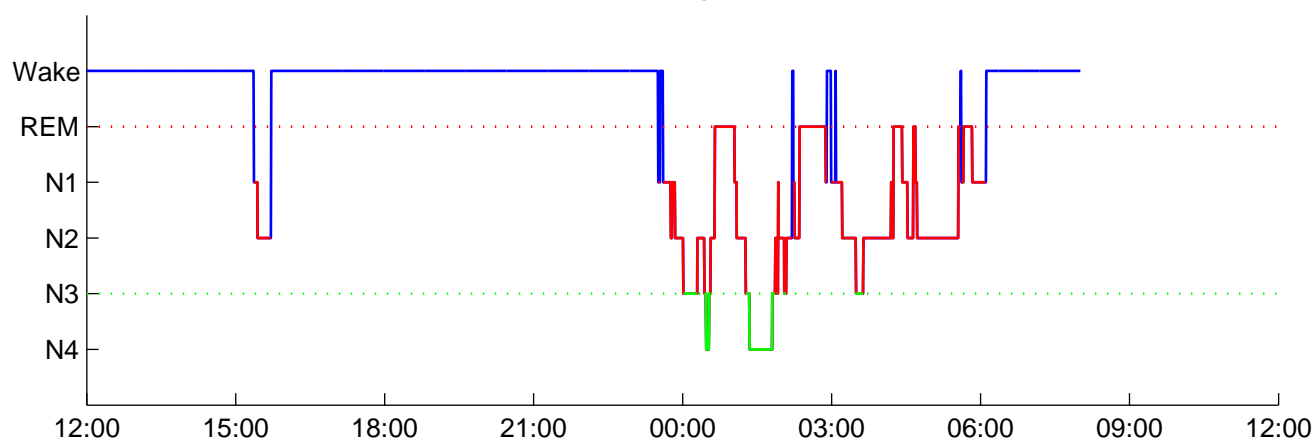

B

IDOS

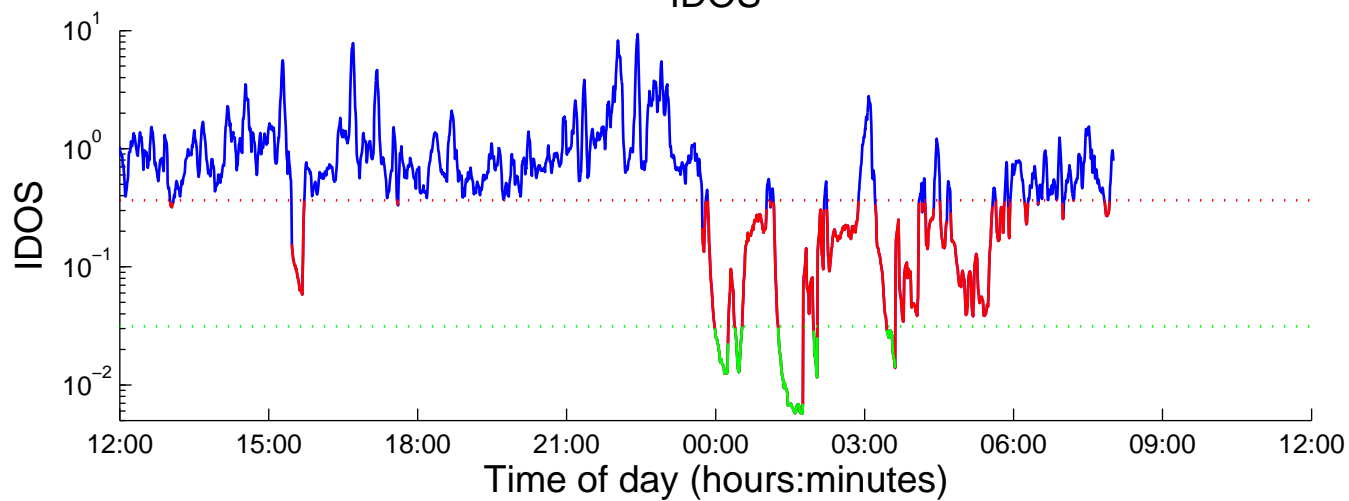

Supplement: Additional file 11 — Hypnogram and IDOS index of outpatient recording 11. The hypnogram resulting from R&K analysis (figure A) of the outpatient recording is shown. The IDOS index for the same recording. The IDOS index (figure B) of the same recording shows similar transitions of depth of sleep. Colors indicate the separation into three classes: wake (blue), non-SWS (red) and SWS (green). The transition from wake to sleep (red dotted line) and from non-SWS to SWS (green dotted line) are also given. The same colors for sleep stages are used for both figures. IDOS, ICU Depth of Sleep; R&K, Rechtschaffen and Kales; SWS, slow wave sleep. [file cc13823-S11.pdf]

A

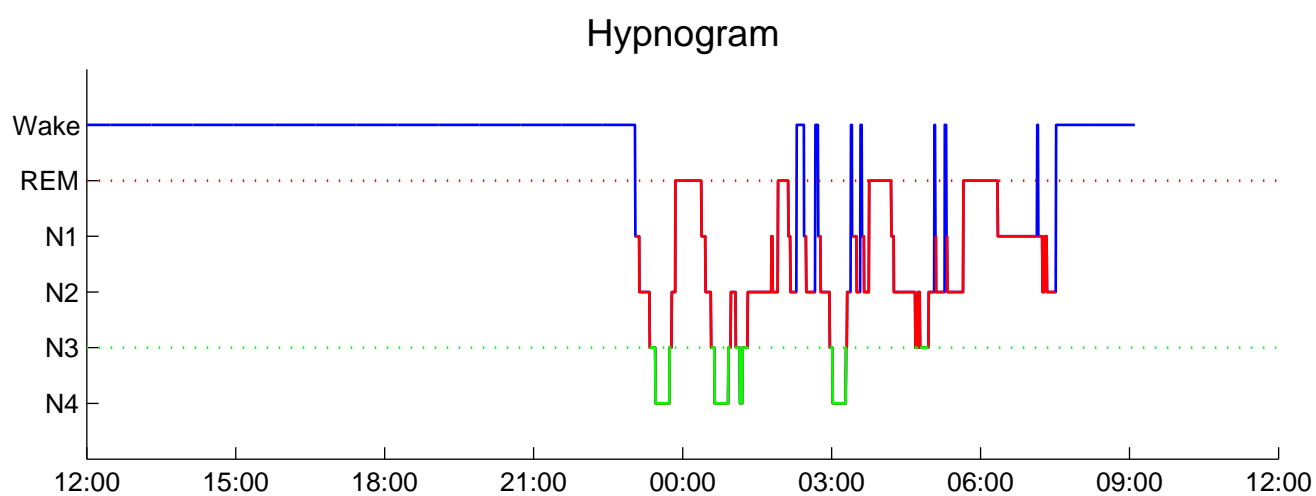

B

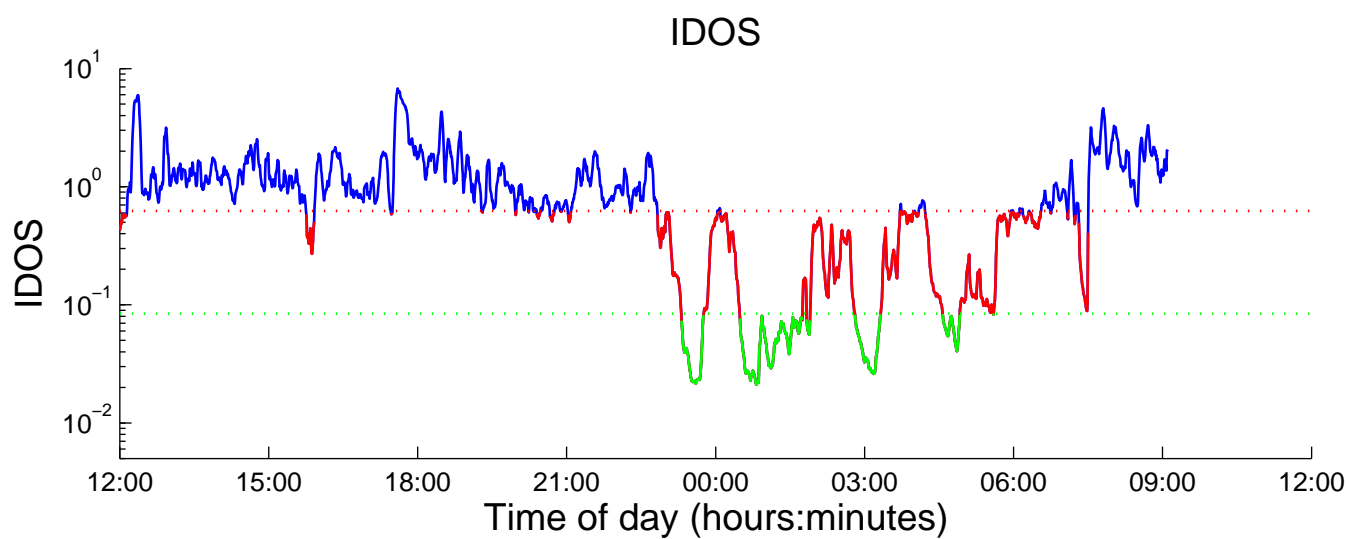

Supplement: Additional file 12 — Hypnogram and IDOS index of outpatient recording 12. The hypnogram resulting from R&K analysis (figure A) of the outpatient recording is shown. The IDOS index for the same recording. The IDOS index (figure B) of the same recording shows similar transitions of depth of sleep. Colors indicate the separation into three classes: wake (blue), non-SWS (red) and SWS (green). The transition from wake to sleep (red dotted line) and from non-SWS to SWS (green dotted line) are also given. The same colors for sleep stages are used for both figures. IDOS, ICU Depth of Sleep; R&K, Rechtschaffen and Kales; SWS, slow wave sleep. [file cc13823-S12.pdf]

A

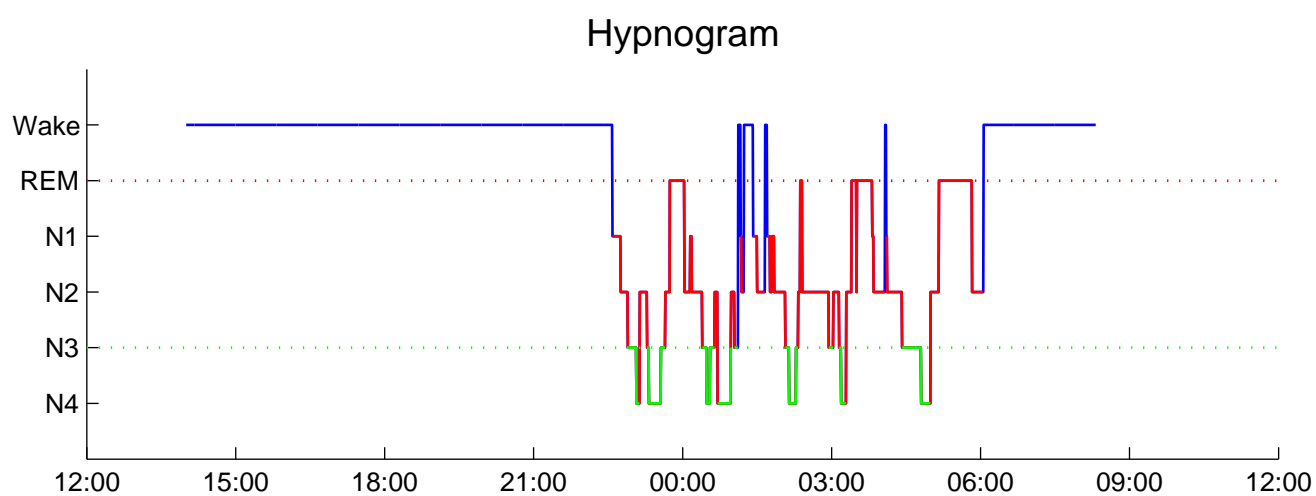

B

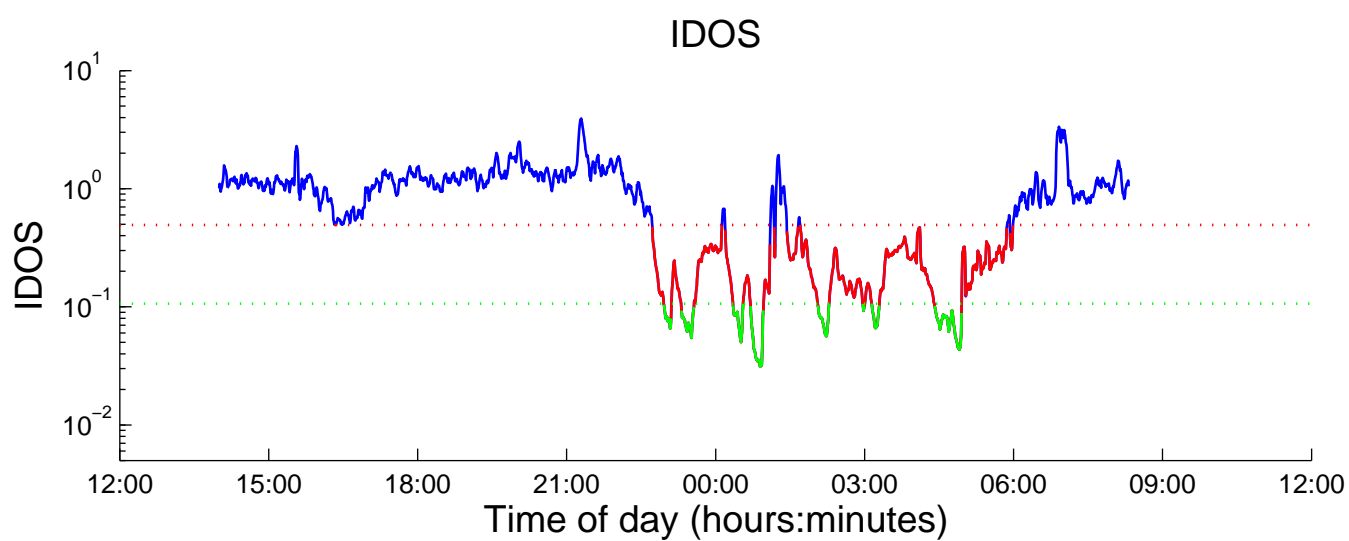

Supplement: Additional file 13 — Hypnogram and IDOS index of outpatient recording 13. The hypnogram resulting from R&K analysis (figure A) of the outpatient recording is shown. The IDOS index for the same recording. The IDOS index (figure B) of the same recording shows similar transitions of depth of sleep. Colors indicate the separation into three classes: wake (blue), non-SWS (red) and SWS (green). The transition from wake to sleep (red dotted line) and from non-SWS to SWS (green dotted line) are also given. The same colors for sleep stages are used for both figures. IDOS, ICU Depth of Sleep; R&K, Rechtschaffen and Kales; SWS, slow wave sleep. [file cc13823-S13.pdf]

A

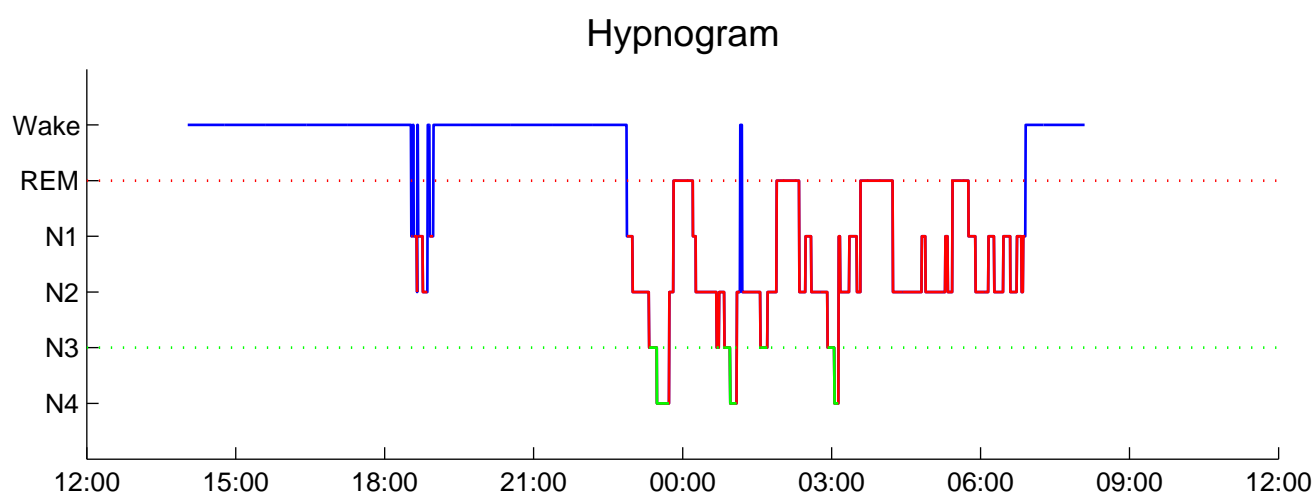

B

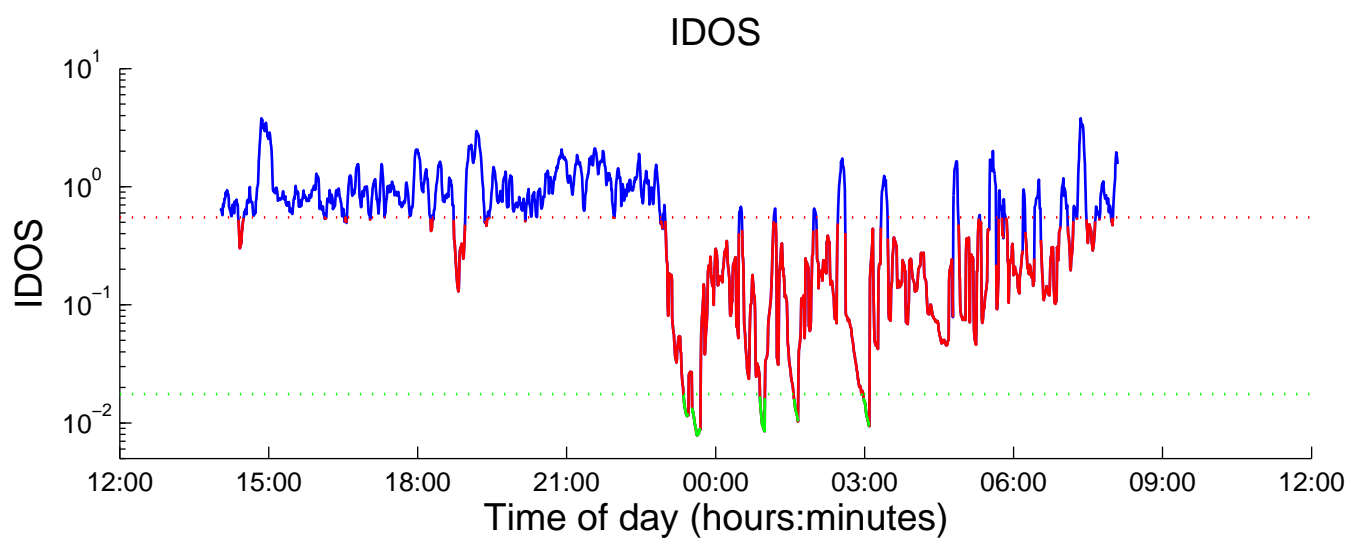

Supplement: Additional file 14 — Hypnogram and IDOS index of outpatient recording 14. The hypnogram resulting from R&K analysis (figure A) of the outpatient recording is shown. The IDOS index for the same recording. The IDOS index (figure B) of the same recording shows similar transitions of depth of sleep. Colors indicate the separation into three classes: wake (blue), non-SWS (red) and SWS (green). The transition from wake to sleep (red dotted line) and from non-SWS to SWS (green dotted line) are also given. The same colors for sleep stages are used for both figures. IDOS, ICU Depth of Sleep; R&K, Rechtschaffen and Kales; SWS, slow wave sleep. [file cc13823-S14.pdf]

A

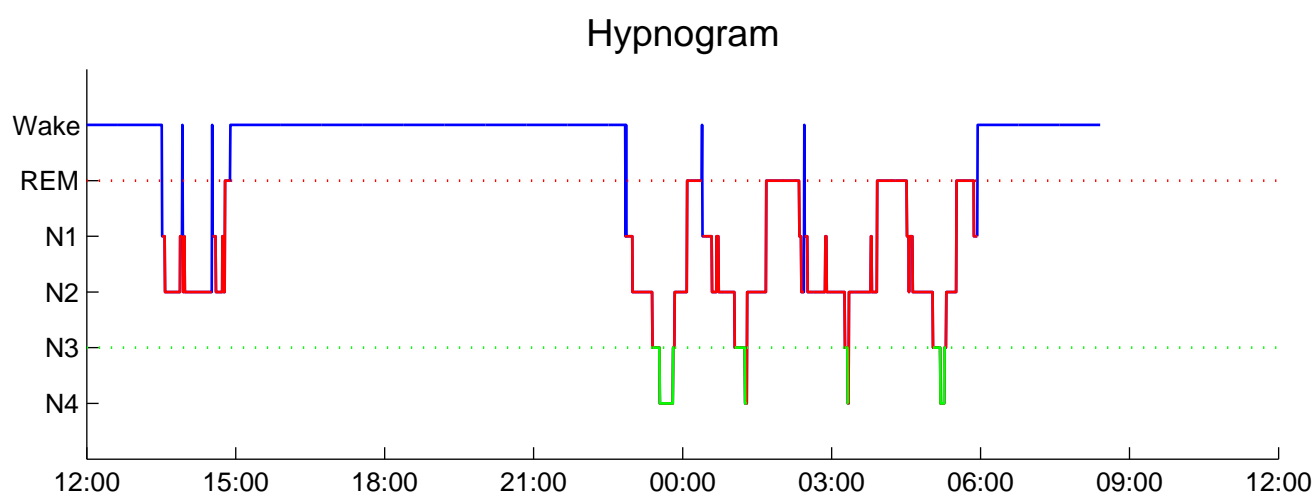

B

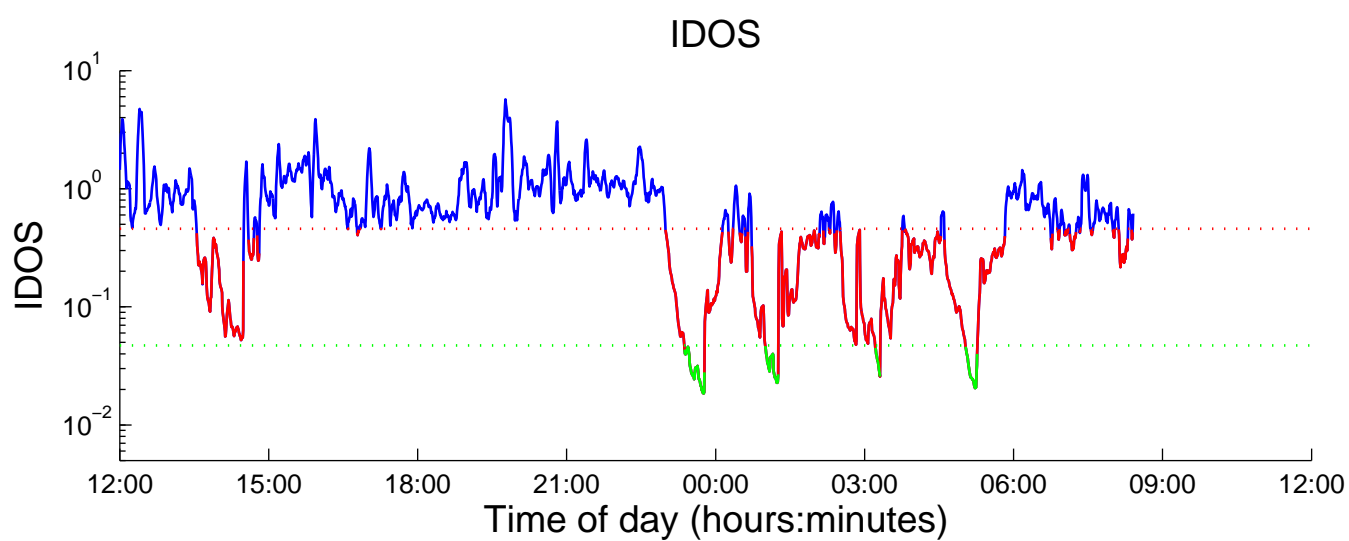

Supplement: Additional file 15 — Hypnogram and IDOS index of outpatient recording 15. The hypnogram resulting from R&K analysis (figure A) of the outpatient recording is shown. The IDOS index for the same recording. The IDOS index (figure B) of the same recording shows similar transitions of depth of sleep. Colors indicate the separation into three classes: wake (blue), non-SWS (red) and SWS (green). The transition from wake to sleep (red dotted line) and from non-SWS to SWS (green dotted line) are also given. The same colors for sleep stages are used for both figures. IDOS, ICU Depth of Sleep; R&K, Rechtschaffen and Kales; SWS, slow wave sleep. [file cc13823-S15.pdf]
